# Transcriptional Profiling and Deriving a Seven-Gene Signature That Discriminates Active and Latent Tuberculosis: An Integrative Bioinformatics Approach

**DOI:** 10.3390/genes13040616

**Published:** 2022-03-29

**Authors:** Sudhakar Natarajan, Mohan Ranganathan, Luke Elizabeth Hanna, Srikanth Tripathy

**Affiliations:** 1 Department of Virology and Biotechnology, ICMR–National Institute for Research in Tuberculosis (NIRT), Chetpet, Chennai 600031, India; mohan.ranganathan85@gmail.com (M.R.); hanna@nirt.res.in (L.E.H.); srikanthtripathy@gmail.com (S.T.); 2 Dr. DY Patil Medical College, Hospital and Research Centre, Pimpri, Pune 411018, India

**Keywords:** tuberculosis, active TB, latent TB infection, differentially expressed genes, biomarkers, bioinformatics

## Abstract

Tuberculosis (TB) is an infectious disease caused by *Mycobacterium tuberculosis* (*M.tb.*). Our integrative analysis aims to identify the transcriptional profiling and gene expression signature that distinguish individuals with active TB (ATB) disease, and those with latent tuberculosis infection (LTBI). In the present study, we reanalyzed a microarray dataset (GSE37250) from GEO database and explored the data for differential gene expression analysis between those with ATB and LTBI derived from Malawi and South African cohorts. We used BRB array tool to distinguish DEGs (differentially expressed genes) between ATB and LTBI. Pathway enrichment analysis of DEGs was performed using DAVID bioinformatics tool. The protein–protein interaction (PPI) network of most upregulated genes was constructed using STRING analysis. We have identified 375 upregulated genes and 152 downregulated genes differentially expressed between ATB and LTBI samples commonly shared among Malawi and South African cohorts. The constructed PPI network was significantly enriched with 76 nodes connected to 151 edges. The enriched GO term/pathways were mainly related to expression of IFN stimulated genes, interleukin-1 production, and NOD-like receptor signaling pathway. Downregulated genes were significantly enriched in the Wnt signaling, B cell development, and B cell receptor signaling pathways. The short-listed DEGs were validated in a microarray data from an independent cohort (GSE19491). ROC curve analysis was done to assess the diagnostic accuracy of the gene signature in discrimination of active and latent tuberculosis. Thus, we have derived a seven-gene signature, which included five upregulated genes *FCGR1B*, *ANKRD22*, *CARD17*, *IFITM3*, *TNFAIP6* and two downregulated genes *FCGBP* and *KLF12*, as a biomarker for discrimination of active and latent tuberculosis. The identified genes have a sensitivity of 80–100% and specificity of 80–95%. Area under the curve (AUC) value of the genes ranged from 0.84 to 1. This seven-gene signature has a high diagnostic accuracy in discrimination of active and latent tuberculosis.

## 1. Introduction

Tuberculosis (TB) is an infectious disease caused by *Mycobacterium tuberculosis* (*M.tb.*). Tuberculosis remains a global health problem and is the leading cause of mortality from a single infectious agent. The World Health Organization (WHO) estimates that 10 million new cases of TB occurred in the year 2019 and 1.4 million people died from TB [1]. Most infections of *M.tb.* manifest as a clinically asymptomatic contained state, known as latent tuberculosis infection (LTBI), which affects one fourth of the global population [2,3]. There is heterogeneity in clinical state of LTBI, which includes individuals who may have eliminated the pathogen, as well as individuals with an incipient or subclinical active disease [4].

Chest X-ray has been popularly used as a screening tool for pulmonary TB. Sputum smear microscopy for acid fast bacilli, and sputum culture, are the commonly used methods worldwide for the diagnosis of pulmonary TB. Sputum culture is the gold standard method for diagnosis of active tuberculosis (ATB), but it takes at least 6–7 days for a positive diagnosis and up to 42 days for a confirmed negative diagnosis, while sputum smear suffers from low sensitivity [5]. The GeneXpert MTB/RIF test and PCR based assays serve as rapid tests for detection of TB but require sophisticated technology and well-trained staff and hence are not affordable in low resource settings [6]. Besides, none of the available sputum-based tests can predict reactivation of TB. The Tuberculin skin test (TST) and Interferon γ release assay (IGRA) available for diagnosis of LTBI cannot accurately differentiate between LTBI and active TB [7].

There is a high risk of latent TB reactivation associated with certain risk factors, such as HIV infection, diabetes, malnutrition, immune suppressive treatment, and active smoking [8]. About 5–10% of latently infected individuals will progress to active TB during their lifetime [9]. Accurate and early diagnosis of active tuberculosis is required to control the TB disease. The World Health Organization’s (WHO) End TB Strategy has set the goals of reducing TB incidence by 90% and TB deaths by 95% globally by 2035 [10]. Therefore, there is an urgent need for development of simple, non-sputum based, highly sensitive, and specific tests for diagnosis of *M.tb.* infection. In this context, blood-based gene expression signatures act as the most sought-after biomarkers for distinguishing individuals with active TB (ATB) disease from those with LTBI. In addition, the prognostic biomarkers that can predict the risk of active TB in individuals with LTBI would be of enormous value, so that preventive drug treatment can be offered [11]. The WHO, in conjunction with Foundation for Innovative Diagnostics (FIND) and working group of the Stop TB Partnership, has published target product profile (TPP) for non-sputum biomarker triage test, and diagnostic and predictive tests for progression of LTBI to ATB disease. The TPPs require a minimum 90% sensitivity and 70% specificity for a triage test, 65% sensitivity and 98% specificity for a diagnostic test [12], and 75% sensitivity and 75% specificity for a test to predict progression from LTBI to active TB disease over a two-year period [13]. Several studies in recent years have found that whole-blood RNA signatures can predict the active TB infection [14,15,16,17] and progression of *M.tb.* infection in persons at risk of developing active TB [18]. 

Transcriptional profiling entails differentially expressed genes, which may have implications in terms of the diagnosis and prognosis of a disease and serve as drug targets. High-throughput methods used for transcriptional profiling include microarray analysis, RNAseq, PCR array, and NanoString. Among these techniques, microarray and RNAseq data are deposited by the researchers in the gene expression omnibus (GEO) database for meta-analysis of data. The raw data from GEO are reanalyzed, annotated, and illustrated in various ways, including gene set enrichment (GSEA) analysis, KEGG (Kyoto Encyclopedia of Genes and Genomes) pathway analysis, reactome pathway analysis, and the STRING—protein–protein interaction network. Thus, transcriptional profiling aids in identifying the differentially expressed genes, associated pathways underlying the biology of the disease, the role of host cellular and immune response, and the cell signaling mechanisms involved in the pathogenesis of TB disease. Our integrative analysis aimed to identify the transcriptional profiling that distinguishes individuals with active TB (ATB) disease, and those with latent tuberculosis infection (LTBI). We have short-listed a seven-gene signature, which included five upregulated genes and two downregulated genes. The short-listed genes were validated using bioinformatics analysis in an independent cohort, which showed a statistically significant difference in gene expression between ATB and LTBI. ROC curve analysis was done to validate the accuracy of the identified biomarkers in discrimination of active and latent tuberculosis.

## 2. Materials and Methods

### 2.1. Data Sources 

Microarray datasets used in this study were retrieved from the National Center for Biotechnology Information’s (NCBI) Gene Expression Omnibus (GEO) database (https://www.ncbi.nlm.nih.gov/geo/ (accessed on 12 March 2019), a public repository for gene expression data [19,20,21]. A search of the GEO profiles related to ATB and LTBI samples in GEO database using the terms “Tuberculosis” [Mesh terms] OR active tuberculosis [All fields] AND “Homo sapiens” [porgn] led to identifying a dataset GSE37250, which is a landmark study conducted by Kaforou et al. Thus, GSE37250 (Platform-GPL10558 Illumina Human HT-12 V4.0 expression bead chip) was utilized for exploring the differential gene expression profile between ATB and LTBI [14]. The study sites chosen by Kaforou et al. were of both urban and rural population in South Africa. Capetown, South Africa has one of the highest TB incidence populations in urban setting, with high rates of HIV infection. Another study site, Karonga district, Northern Malawi, is a rural setting, which has comparatively low TB incidence rate with stable HIV prevalence [14]. 

The actual dataset GSE37250 includes data on samples from those with active tuberculosis with/without HIV infection, latent tuberculosis with/without HIV infection, and other diseases with/without HIV infection from Malawi and South African population. In the present study, we downloaded and reanalyzed 51 ATB samples and 35 LTBI samples both without HIV co-infection from Malawi cohort and 46 ATB samples and 48 LTBI samples both without HIV co-infection from South African cohort. 

### 2.2. Data Processing and Differential Gene Expression Analysis

BRB array tool is an analytic and visualization tool integrated into excel so as to visualize and statistically analyze the microarray gene expression data. We used BRB-Array (V 4.6.0, stable version) class comparison tool [22] to identify the DEGs (differentially expressed genes) in peripheral blood of ATB and LTBI from Malawi and South African cohort, respectively. The GSE37250 data file [14] was imported and processed through spot filtering, normalization, and gene-filtering criteria (gene exclusion criteria ≤ 1.5-fold change and expression data value less than 20%). Class comparison analysis was performed between ATB and LTBI classes, and multivariate permutation test was computed based on 1000 random permutations and a false discovery rate of 1%. After identifying the DEGs while comparing ATB vs. LTBI from Malawi cohort and DEGs in South African cohort, we intended to list the common upregulated genes and downregulated genes from the respective cohorts, and these differentially expressed common genes were illustrated using VENNY online web server (https://bioinfogp.cnb.csic.es/tools/venny/, accessed on 15 March 2019). Heatmap of differentially expressed upregulated and downregulated genes was illustrated using the online tool heatmapper (http://www.heatmapper.ca/, accessed on 18 March 2019) [23]. Volcano plot was used to display the statistically significant genes in the form of a scatter plot. Volcano plot was constructed using the −log10 *p* value of differentially expressed upregulated and downregulated genes in Y-axis versus log2 fold change of the DEGs in X-axis. The volcano plot of the DEGs was prepared and presented using Microsoft excel 2010 (ver 14.0).

#### 2.2.1. Gene Ontology and Pathway Enrichment Analysis of Top DEGs

The most upregulated and downregulated genes were further analyzed using the Database for Annotation, Visualization and Integrated Discovery (DAVID) bioinformatics tool (https://david.ncifcrf.gov/tools.jsp, accessed on 20 March 2019). DAVID is a web-based program that investigates and extracts the biological meaning from a large list of genes [24]. In this study, functional annotation, overrepresented Gene Ontology (GO), and KEGG (Kyoto Encyclopedia of Genes and Genomes) pathways identified genes with *p* value < 0.05, and enrichment scores ≥ 3 were shortlisted. Reactome pathway analysis (https://reactome.org/, accessed on 21 March 2019) [25] was performed to identify genes participating in a network of biological interaction/pathways. A *p* value < 0.05 was considered to be statistically significant. The functional annotation of DEGs and pathway analysis have been reported earlier in various diseases [26,27,28,29].

#### 2.2.2. Analysis of Protein–Protein Interaction Network

Protein–protein interaction (PPI) network of most upregulated genes was constructed using the STRING online database (https://string-db.org/, accessed on 21 March 2019) [30] for predicting protein–protein interactions, including direct (physical) and indirect (functional) associations. The resulting network was imported in cytoscape software (https://cytoscape.org/, accessed on 22 March 2019) [31]; Mcode clustering method was used to find closely associated regions in this network, and two densely connected clusters were identified with mcode score > 3.2. MCODE analysis was performed with default parameter; degree cutoff = 2, node score cutoff = 0.2, k-core  =  2, and max. depth  =  100. Important hub genes in the PPI network were identified using cytohubba plugin with Maximal Clique Centrality (MCC) method, and the top 20 hub genes were ranked based on the highest MCC score. Functionally enriched PPI subnetworks were created using ClueGO/CluePedia plugin from cytoscape [32,33], and statistical parameters were maintained as two-sided hypergeometric test with a Benjamini–Hochberg corrected *p* value ≤ 0.05 and kappa scores ≥ 0.4 as criteria. ClueGO was used to visualize the biological terms of large gene clusters [33]. 

### 2.3. Validation of DEGs in An Independent Cohort Using Bioinformatics Analysis

The DEGs identified from Malawi and South African cohorts of active TB and latent TB infection were short listed and based on significant expression and fold change, and the genes were validated in an independent cohort. We searched the GEO database using the terms “Tuberculosis” [mesh terms] OR active tuberculosis [all fields] AND “Homo sapiens” [porgn] and chosen the dataset GSE19491 (https://www.ncbi.nlm.nih.gov/geo/query/acc.cgi?acc=GSE19491, accessed on 24 November 2021) [34] for validation of DEGs in an independent cohort. We randomly selected 20 samples of ATB and 20 samples of LTBI derived from geographic population comprised of South African, and UK cohorts. Datasets from ATB samples before starting treatment were selected for analysis. GEO2R tool was utilized to identify the differentially expressed genes between ATB and LTBI. The GEO2R performs differential expression analysis using GEOquery and Limma R packages from Bioconductor project [21,35]. The GSE19491 dataset was analyzed with default setup. Sequence of data identification information was annotated with NCBI generated annotation or submitter supplied annotation platforms, and quantile normalization was applied to the identical value distribution. In the GEO2R, samples of ATB and LTB were assigned to the respective group for analysis. Significant level cutoff *p* value < 0.05, log_2_ fold change >1 was applied to distinguish the differentially expressed genes in ATB vs. LTBI, and analysis was performed. The analysis listed the top 250 differentially expressed genes with log_2_ fold change (FC) and *p* value, and the genes were ranked by significance. 

Gene expression profile of a particular gene was visualized by clicking on a gene ID. Each red bar in the graph represented the expression measurement extracted from the original submitter supplied sample record. The sample values denoting the expression were converted into log_2_. The log_2_ values were denoted in a box plot for representation.

### 2.4. Receiver Operating Characteristic Curve Analysis

Receiver operating characteristic (ROC)/area under the curve (AUC) analysis was prepared by choosing log2 expression of LTBI samples as control and ATB samples as test using GraphPad prism 8.0. The accuracy and performance of each single biomarker gene were measured by calculating area under the curve (AUC) values. Optimal cutoff values were selected, and the sensitivity and specificity of each biomarker were identified from the graph.

### 2.5. Statistical Analysis

A *p* value of < 0.05 was considered to be statistically significant in short listing the DEGs. In the case of GEO2R analysis, the log_2_ expression value of ATB and LTBI samples were illustrated in box plot, and the statistical significance was calculated using non-parametric Mann–Whitney U test. Statistical analysis and ROC/AUC analysis were done using GraphPad prism 8.0.

## 3. Results

### 3.1. Identification of Top Significant DEGs Derived from Microarray Dataset GSE37250

We downloaded the gene expression dataset (GSE37250) comprised of microarray data from ATB and LTBI samples from GEO database, based on GPL10558 platform (Illumina Human HT-12V4.0 expression bead chip). Class comparison analysis between ATB and LTBI groups identified 591 DEGs in Malawi cohort and 962 DEGs in South African cohort. 

Heatmap of upregulated genes is presented in Figure S1, which shows the distribution of genes among three major clusters. Cluster 1 includes *CR1, KREMEN1, FCGR2C, P2RY14, IGF2BP3, CARD17,* and *ZAK* genes; Cluster 2 includes *GAS6, TLR2, SMARCD3, GPR84, GBP6, CASP5, HIST2H2A, C1QC, FCAR, CLEC4D, MAPK14, APOL6, HP, C1Qb, BATF2, DEFA4,* and *CEACAM8;* and Cluster 3 includes *JAK2, CEACAM1, CARD16, CACNA1E, ANKRD22, FCGR1B, SIGLEC5, SLP1, TNFAIP6, AIM2, FCGR1C, FCGR1A, IFIT3, SERPING, DEFA3, DEFA1B, GBP5,* and *GBP1.*

Heatmap of downregulated genes in Figure S2 illustrate the distribution of genes among four major clusters. Cluster 1 includes *RPL31P43, WNT7A, HAUS5, LINC01550, CLIC5, RTKN, C2orf40,* and *DKK3; cluster 2* includes *COL9A2, HABP4, CEP68, AEBP1, IL23A, TCF7, CSNK1E, DTX3, MAP4K1, TLE2, PKD1,* and *ANO9; cluster 3 includes HPCAL4, NDRG2, LRRC26, MEGF6, EPHA4, LAMA5, EPHB6, NFATC3, IL11RA, PLXNA3, SIRPG, SAMD3, GPR68, KLF12, TCL1A,* and *CD8B;* and cluster 4 includes *CRIP2, EBF1, OSBPL10, LINC00926, BLK, ID3, NCR3, CXCR3, LRRN3, FCGBP, CD19, FCRLA, CXCR5, TNFRSF25, CD27,* and *GZMK.*

**Figure 1:**
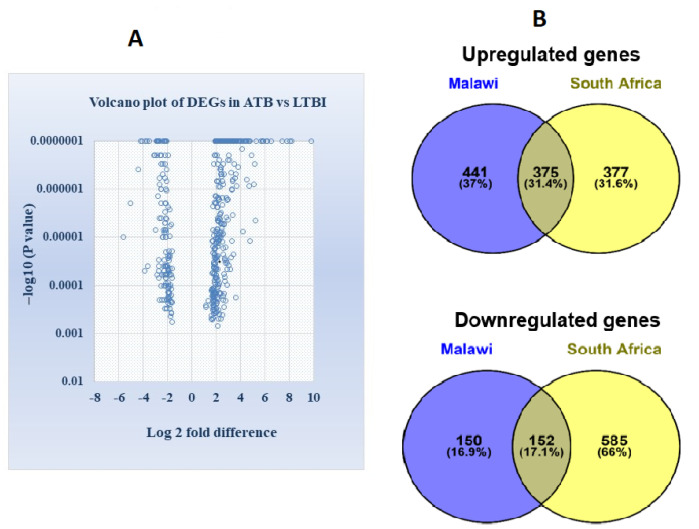
(**A**) Volcano plot illustrating the identification of differentially expressed genes between ATB and LTBI in the Malawi and South African cohort (differentially expressed genes were distinguished with parameter −log10 *p* value in y axis and log2 fold change in x axis). Significant results were determined based on cut off range, *p* value < 0.01 and >1.5-fold change (**B**) Venn diagram demonstrating the intersection of differentially expressed overlapped or common genes, in Malawi and South African cohort derived from GSE37250 (Figure S1).

Volcano plot showing the distribution of statistically significant DEGs at *p* value < 0.01 and fold change > 1.5 as gene filtering criteria are illustrated in Figure 1A. Differentially expressed overlapping genes common in Malawi and South African cohorts of ATB and LTBI groups were identified. Venn diagram in Figure 1B displays 375 upregulated genes and 152 downregulated genes commonly shared among the two cohorts. 

*FCGR1A, FCGR1B, FCGR1C, BATF2, GBP1, ANKRD22, GBP5, AIM2, GBP6,* and *CASP4,* were ranked among the top overexpressed genes in ATB compared to LTBI, whereas *NDRG2, KLF12, ANO9, CD79A,* *FCGBP,* *GZMK,* *CXCR3, MIEF2, CXCR5,* and *CD27* genes were the most downregulated genes in active TB vs. LTBI. The top 10 upregulated and downregulated genes of ATB and LTBI from Malawi and South African cohorts are presented in Table 1A,B. The complete list of most upregulated and downregulated genes is presented in Supplementary Table S1.

### 3.2. Pathway Enrichment and Functional Annotation of Top DEGs

We have investigated the biological function of the top DEGs using GO, KEGG, and Reactome pathway analysis tools available in the DAVID online bioinformatics tool (modified Fishers exact *p*-value (EASE score) ≤ 0.05 was considered significant in the GO term/pathway). GO biological pathway analysis revealed that the upregulated genes were associated with innate immune response, leukocyte migration, antibacterial humoral response, defense response to bacterium, inflammatory response, platelet degranulation, killing of cells of other organisms, and regulation of apoptotic process (Table 2). On the other hand, downregulated genes were mainly related to Wnt signaling pathway, cell differentiation, inflammatory response, regulation of cell proliferation, and B cell receptor signaling pathway (Table 3). The most enriched KEGG pathways of upregulated genes were involved in *Staphylococcus aureus* infection, leishmaniasis, tuberculosis, systemic lupus erythematosus, complement and coagulation cascade, and phagosome formation (Table 2). Downregulated genes were significantly enriched in primary immunodeficiency, cytokine–cytokine receptor interaction, hematopoietic cell lineage, Wnt signaling, and B cell receptor signaling pathway (Table 3).

Reactome pathway analysis of the upregulated genes revealed that neutrophil degranulation, immune response, interferon signaling, interferon γ signaling, fibronectin matrix formation, RMT methylate histone arginines, α defensins, and cytokine signaling in immune system were significantly enriched (Table 2). Additionally, *WNT* target genes, with *TNF* binding to their physiological receptor, centrosome maturation, and antigen activated B cell receptor, were significantly downregulated (Table 3).

### 3.3. Analysis of Protein–Protein Interaction (PPI) Network among the DEGs and Identification of Hub Genes for the Upregulated Gene Network

**Figure 2:**
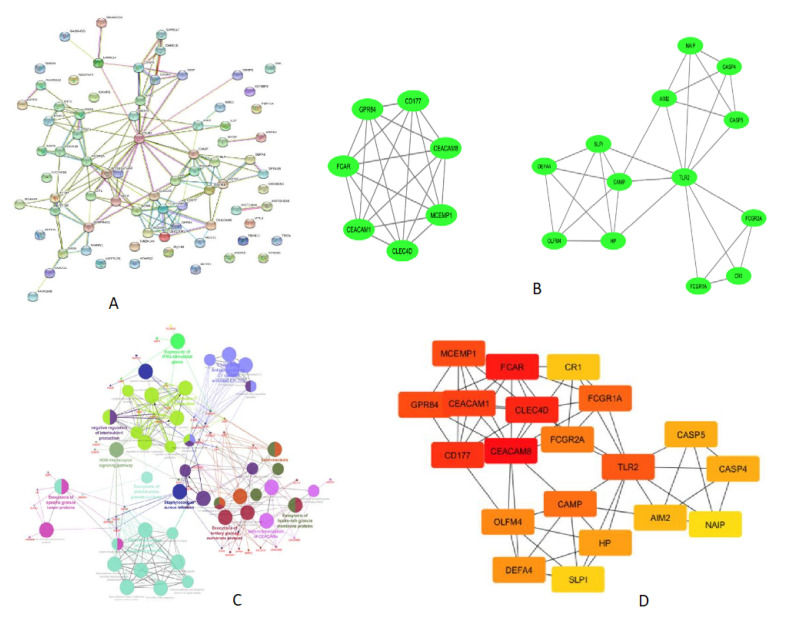
PPI network displaying the interaction of proteins coded by the upregulated genes derived from ATB vs. LTBI data. (**A**) Results of STRING analysis (*p* value < 1 × 10^−16^). PPI network with 76 nodes connected to 151 edges. (**B**) Closely connected subnetworks identified by MCODE analysis plugin of cytoscape. Two clusters enriched in top with cluster score above 3 are shown. (**C**) Functionally enriched edges identified from PPI network using clueGO/cluepedia of cytoscape software. Network connectivity among GO term and pathway determined based on the interaction of functional cluster, edges (kappa score > 0.4), and enriched terms/pathway with *p* value < 0.05. Functional groups are denoted in different color codes; the most enriched functional term is indicated in bold color. (**D**) Cytohubba (MCC method) analysis explored the most important hub nodes; nodes in red color indicate a high MCC score, and yellow color node represents a low MCC score.

The protein–protein interaction (PPI) network analysis of most upregulated genes was performed using STRING software tool to examine the functional interaction between the upregulated genes and identify the hub genes in the network. The resulting PPI network was significantly (*p* value < 1 × 10^−16^) enriched with 76 nodes connected to 151 edges (Figure 2A). Molecular interaction of closely associated nodes inferred with confidence level 0.6–0.9 (combined score) found that loosely associated nodes interacting each other were arranged in groups with a combined score of 0.9. The hub nodes *CLEC4D, CEACAM8, CEACAM1, CEACAM6,* *GPR84, FCAR,* and *MCEMP1* were interacting with each other and connected with 21 edges with a MCODE score of 7. The other 6 nodes *OLFM4, DEFA4, SLPI, HP, CAMP,* and *TNFAIP6* were interconnected with each other (Figure 2B). Another six nodes, *FCGR1B, FCGR1A, GBP1, GBP5, DBP6,* and *IFIT3,* were interconnected in a group with a combined score 0.7–0.9. The functional interaction of PPI network was further analyzed in detail using the ClueGO/CluePedia plugin of cytoscape. 

The enriched GO term/pathways were mainly related to expression of *IFNG* stimulated genes (*FCGR1A, FCGR1B, GBP6, GBP5,* and *GBP1*), antigen-antibody C1 complex activated C1r, C1s (*C1QC, C1QB, SERPING1, MAPK14,* and *CR1*), interleukin-1 production (*AIM2, GBP5, GBP1, GBP6, CARD17, CARD16, CEACAM1, SERPING1,* and *JAK2*), NOD-like receptor signaling pathway (*GBP1, AIM2, CARD17, GBP5, CARD16, MAPK14, CASP5, CASP4, NAIP, DEFA1B, DEFA3,* and *DEFA4*), HNP1–4 stored in primary neutrophil granules (*NRG1, DEFA4, DEFA3, DEFA1B,* and *CAMP*), exocytosis of tertiary granule membrane proteins (*FCAR, MCEMP1, GPR84, SINGLEC5, CEACAM8, CD177, CLEC4D,* and *CR1*), exocytosis of ficolin rich granule membrane proteins (*CR1, TLR2,* and *SELP*), and exocytosis of specific granule lumen proteins (*SLPI, HP, OLFM4, CAMP, DEFA3, DEFA4,* and *TNFAIP6*) (Figure 2C).

### 3.4. Gene Ontology and Pathway Analysis of Downregulated Genes

The enriched GO term/pathway analysis revealed that the downregulated genes were primarily involved in Wnt signaling (*WNT7A, CSNK1E, DKK3, TCF7,* and *TLE2)*, *CD19, CD79A, CD8B,* and *TNFRSF13C),* primary immunodeficiency (*CD19, CD79A, CD8B,* and *TNFRSF13C),* immune response (*CXCR5, CD27, CD8B, TNFRSF25, NCR3, VPREB3,* and *TCF7),* cytokine-cytokine receptor interaction (*CXCR3, CXCR5, CD27, TNFRSF13C, TNFRSF25, IL11RA,* and *IL23A),* regulation of cell proliferation (*BLK, CD27, TNFRSF25, LAMA5,* and *TCF7),* and B cell receptor signaling pathway (*BLK, CD19,* and *CD79A)* (Figure 2C).

MCODE analysis of cytoscape found the highly interconnected region of PPI network, CLEC4D, GPR84, CEACAM8, CEACAM1, CD177, FCAR, and MCEMP1 are the seven nodes that were highly interlinked with a MCODE score 7. The other 13 nodes include CASP4, DEFA4, CAMP, FCGR1A, TLR2, CASP5, CR1, AIM2, SLPI, NAIP, OLFM4, FCGR2A, and HP, which are interlinked with a MCODE Score 5. TLR2 is one such hub node, which is interconnected with other nodes. The details of interlinked nodes and their MCODE score in each module are represented in Table 4A. MCC method of cytohubba plugin recognized maximum centrality among nodes in the network. A total of 19 hub nodes presents the closest connection with other nodes (Figure 2D). The top-ranked nodes and their MCC scores are shown in Table 4B.

### 3.5. Deriving and Validation of a Seven-Gene Signature in Discrimination of Active and Latent Tuberculosis 

We aimed to deduce a short signature including both upregulated and downregulated genes for discriminating active and latent tuberculosis infection. Differential gene expression analysis using GEO2R analysis was done by randomly selecting 20 samples of ATB and 20 samples of LTBI from an independent cohort (GSE19491). Based on fold change of the most differentially expressed genes in Malawi and South African cohort (Table 1A), the top DEGs were shortlisted and validated in an independent cohort. The expression and fold change of top DEGs in the independent cohort were *FCGR1B* (3.6-fold up), *FCGR1A* (2.8-fold up), *ANKRD22 (4.0-fold up)*, *GBP5* (2.5-fold up), *GBP1* (2.1-fold up), *BATF2* (3.1-fold up), *CARD17* (3.6-fold up), *CASP4* (1.0-fold up), and *TNFAIP6* (1.97-fold up). 

The expression of top hub genes (Table 4B) was also checked in the independent cohort. The expression of hub genes was as follows: *CEACAM8* (0.97-fold up), *CEACAM1* (1.82-fold up), *CLEC4D* (2.0-fold up), *MCEMP1* (1.40-fold up), *GPR84* (2.1-fold up), and *TLR2* (0.47-fold up).

After obtaining these results, the dataset from 20 randomized samples of ATB and LTBI, respectively, was chosen again from the validation cohort, and the DGE expression was performed using GEO2R. This was repeated twice. The differentially expressed genes, which were consistently expressed in the independent cohort, were chosen. 

Thus, the most differentially expressed upregulated genes *FCGR1B, ANKRD22, CARD17, TNFAIP6*, and *IFITM3* were selected by verifying the consistency of expression in an independent cohort (GSE19491). 

The most downregulated genes in ATB vs. LTBI (Table 1B) were validated in the independent cohort GSE19491, and the expression was as follows: *NDRG2* (1.4-fold down), *KLF12* (1.2-fold down), *ANO9* (1.1-fold down), *CD79A* (1.1-fold down), *FCGBP* (2.2-fold down), *GZMK* (1.3-fold down), *CXCR3* (1.3-fold down), *CXCR5* (1.5-fold down), and *CD27* (0.9-fold down). Among the downregulated genes, *KLF12* (1.2-fold down) and *FCGBP* (2.2-fold down) were selected based on fold change and consistency of expression in the independent cohort.

**Figure 3:**
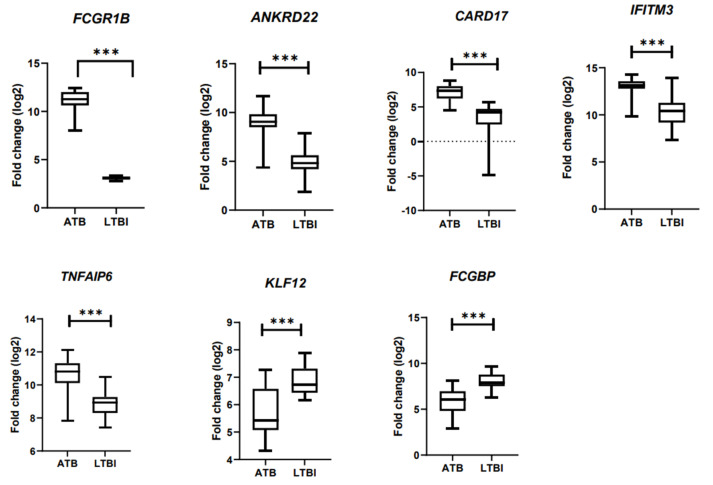
Validation of DEG in an independent cohort using bioinformatics analysis. *** *p* < 0.001.

**Figure 4:**
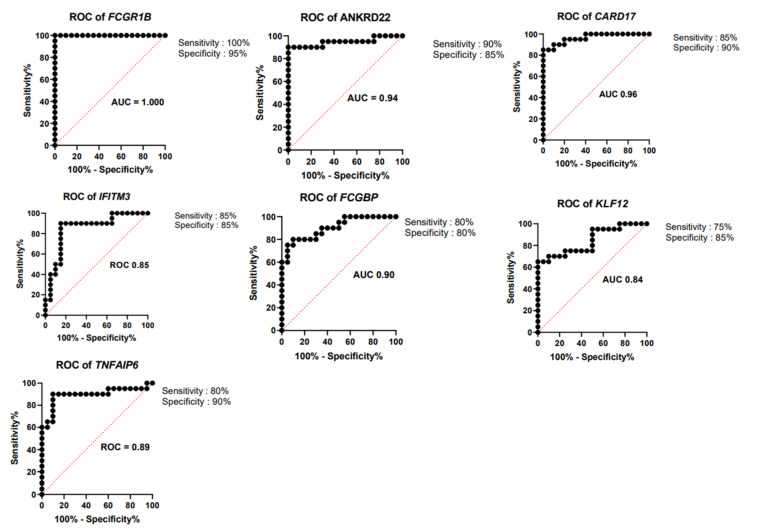
Receiver operating characteristic curve (ROC) analysis. ROC curves for upregulated genes *FCGR1B, ANKRD22, CARD17, IFITM3,* and *TNFAIP6* and downregulated genes *FCGBP* and *KLF12*.

Thus, we have derived a seven-gene signature, which included five upregulated genes *FCGR1B, ANKRD22, CARD17, IFITM3,* and *TNFAIP6* and two downregulated genes *FCGBP* and *KLF12,* as a diagnostic biomarker for discrimination of active and latent tuberculosis infection. The short-listed genes were validated in an independent cohort, through bioinformatics analysis, which showed a statistically significant difference in gene expression between ATB and LTBI samples (Figure 3). Hence, we derived a seven-gene signature and the accuracy of the identified biomarkers was validated in discrimination of active and latent tuberculosis. The identified genes have a sensitivity of 80–100% and specificity of 80–95% (Figure 4). 

The seven-gene signature included five upregulated genes *ANKRD22* (ankyrin repeat domain containing protein 22), *CARD17* (caspase recruitment domain containing protein 17), *IFITM3* (interferon-induced transmembrane protein 3), *TNFAIP6* (TNF α Induced Protein 6), two downregulated genes *FCGBP* (Fc γ binding protein), and *KLF12* (Kruppel Like Factor 12). The short-listed genes were validated in an independent cohort, through bioinformatics analysis, which showed a statistically significant difference in gene expression between ATB and LTBI samples

## 4. Discussion 

Whole-blood-gene expression profiling among active and latent TB-infected individuals can identify a wide range of potential transcriptional biomarkers for active TB diagnosis. Several host–response-based signatures have been reported over the last decade for distinguishing patients with ATB from those with LTBI, other diseases, and uninfected healthy controls [14,18,36,37]. In the present study, we reanalyzed the transcriptional profile of ATB and LTBI in microarray datasets derived from Malawi and South African cohorts [14]. Our analysis identified 375 upregulated genes and 152 downregulated genes that were common and consistently expressed in both the cohorts (Figure 1). Since only the common or overlapping differentially expressed genes in ATB vs. LTBI were explored for analysis, the DEGs identified can be used as a biomarker in a population with high incidence as well as low incidence of tuberculosis.

Gene ontology/pathway analysis helps us to understand the pathways involved in active tuberculosis. Blood transcriptional profiling of ATB vs. LTBI revealed that interferon signaling genes (*FCGR1A* and *FCGR1B*) were predominantly upregulated during active tuberculosis as reported in earlier studies [34,38]. Significantly enriched gene sets in the Gambian cohort study (ATB vs. LTBI) were involved in systemic lupus erythematosus, complement coagulation cascade, and Fc γ receptor-mediated phagocytosis [36]. Using RNAseq-based gene expression profiling, Estevez et al. showed that expression of genes related to neutrophil degranulation, interferon γ signaling, complement cascade, interferon (type I and type II) signaling, and antimicrobial peptide genes is highly activated in ATB compared to LTBI [39]. Thus, our gene-expression profiling and functional pathway analysis observed a compiled and unique pattern of gene expression signatures reported in previous studies. In accordance with earlier studies, the genes corresponding to immune response and to defense response to bacterium were enriched. On the other hand, downregulated genes were coding for proteins involved in Wnt signaling, B cell receptor signaling, primary immunodeficiency, cytokine–cytokine receptor interaction, and cell differentiation. Our results corroborate with earlier studies that show that key genes participating in Wnt signaling pathway were impaired in severe pulmonary TB patients [40] and B cell and T cell transcript signatures were decreased in active tuberculosis [36,41]. 

PPI network analysis of DEGs identified groups of closely interconnected nodes in the upregulated network (Figure 2A), and the ClueGO/Cluepedia analysis predicted the functional interpretation of this closely interacted nodes. The resulted ClueGO terms were related to complement, interferon (expression of IFN stimulated genes), and NOD-like receptor signaling pathway, which were reported in previous studies done elsewhere [34,37,38,42,43]. In addition, we observed significant enrichment of HNP1–4 stored in primary neutrophil granules, exocytosis of membrane protein, exocytosis of lumen protein, and heterodimerization of CEACAMs (Figure 2C). 

MCODE cluster and MCC cytohubba analysis identified densely connected hub genes in the network (Figure 2B). Our results demonstrate that the hub genes are predominantly active in tuberculosis and are involved in host response to *Mycobacterium tuberculosis*. The crucial role of FCGRs in antigen uptake is influenced by highly activating Fc receptor for IgG and immune complex [44]. CLEC4D, CEACAM8, CEACAM1, CEACAM6, GPR84, FCAR, and MCEMP1 are the top hub nodes or proteins tightly connected with other nodes in the active TB (Figure 2B). *CD177* and *CEACAM8* genes are responsible for neutrophil activation in active TB, and expression of CD66a is increased upon mycobacterial infection in a time- dependent manner [45]. The C-type lectin receptor Clecsf8/clec4d is a key component in anti-mycobacterial host defense [46]. *CEACAM1, CEACAM6*, and *CEACAM8* genes were expressed >3-fold in active TB. *CEACAM1* (CD66a), *CEACAM6* (CD66c), and *CEACAM8* (CD66b) are genes coding for carcinoembryonic antigens of the immunoglobulin superfamily. Soluble recombinant *CEACAM8*-Fc dampens the *TLR2*-triggered immune response by interacting with *CEACAM1* expressing human airway epithelium [47]. *AIM2* was one of the top 10 genes, which is upregulated 4.1-fold in ATB compared to LTBI. *AIM2* senses cytosolic double-stranded DNA (dsDNA), which activates the inflammasome host immune response to pathogens. *AIM2*-deficient mice showed increased susceptibility to *M.tb.* infection due to a defect in the production of IL−1β, IL18, and impaired Th1 response [48]. *TLR2* gene was among the top 30 genes upregulated in ATB. TLR2 is a hub node interlinked with *FCGR1A, CAMP, FCGR2A, HP, CASP4, CASP5, AIM2, CR1, SLPI, NAIP, OLFM4*, and *DEFA4* (Figure 2B). Toll-like receptor 2 (TLR2), expressed on the apical surface of airway epithelial cells, is particularly important for the detection of inhaled bacteria in the human airways and for the initiation of the innate immune response [49]. TLR2 signaling is highly regulated during *M.tb* infection and plays a protective multi-faceted role in containing chronic *M.tb* infection [50]. 

Majority of the enlisted upregulated and downregulated genes in our study are consistent with the 27 transcript signatures identified by the original submitter Kaforou et al. for distinguishing TB from latent TB infection [14,16]. Our results also corroborate a UK study by Blankley et al. that identified these genes within the top 10 upregulated genes in pulmonary TB patients as compared to healthy controls [16]. Maertzdorf et al. derived a combination of five most prominently differentiating genes *FCGR1B, CD64, LTF, GBP5*, and *GZMA* as a biosignature for TB diagnosis. This five-gene biosignature yielded the highest accuracy in discriminating between ATB and LTBI, with a sensitivity and specificity of 94% and 97%, respectively [36]. A 16-gene signature was identified in a study by Zak et al. that comprised genes *ANKRD22, APOL1, BATF2, ETV7, FCGR1A, FCGR1B, GBP1, GBP2, GBP4, GBP5, SCARF1, SEPT4, SERPING1, STAT1, TAP1*, and *TRAFD1* [18]. A meta-analysis performed with 16 microarray datasets to profile the host transcriptional response in active tuberculosis led to the identification of five upregulated genes: *AIM2, BATF2, FCGR1B, HP,* and *TLR5* [16]. Sweeney et al., in an integrated multi-cohort analysis, discovered a blood-based three-gene signature, *GBP5*, *DUSP3,* and *KLF2*, that distinguish patients with ATB from healthy controls, as well as from those with LTBI and other diseases [17]. The same group later evidenced that the three-gene TB score was significantly associated with progression of individuals from LTBI to ATB, six months prior to a sputum positive test result [51]. Of note, the differentially expressed upregulated genes such as *BATF2, C1QB, CAMP, CASP5, FCGR1A, FCGR1B, FCGR1C, GBP1, GBP6, IFIT3,* and *P2RY14*, and the downregulated genes *CD19, CD27, CD79A, CXCR3, CXCR5, GZMK, TCF7, ID3,* and *TCF7,* derived from Malawi and South African cohorts, were consistent and comparable with blood transcriptome profile of tuberculosis and tuberculosis-diabetes co-morbidity studied in Indian population [52]. 

The differentially expressed common genes identified from Malawi and South African cohorts of active TB and latent TB infection were short listed and based on significant expression and fold change, and the DEGs were validated in an independent cohort. The seven-gene signatures were selected majorly based on fold change of the most differentially expressed genes. Though both *FCGR1A* and *FCGR1B* were consistently expressed in validation cohort, we have chosen only one gene (*FCGR1B*) from FCGR family of genes. The other top DEGs such as *GBP5*, *GBP1*, and *BATF2* were consistently expressed, but these genes were part of known signatures genes reported earlier [16,17,18]; hence, they were not included in deriving the gene signature. Based on fold change of the most differentially expressed genes, *FCGR1B, ANKRD22, CARD17, TNFAIP6,* and *IFITM3* were short-listed, and these genes were finalized by consistent expression of these genes in an independent cohort. We tried to incorporate the hub genes such as *TLR2* and *CEACAM* family of genes in deriving the signature. However, *TLR2* gene was not consistently expressed in the validation cohort, whereas *CEACAM1* and *CEACAM8* genes were expressed in validation cohort but the expression level was low. Hence, *CEACAM1* and *CEACAM8* genes were not preferred in the signature genes. The downregulated genes *KLF12* (1.2-fold down) and *FCGBP* (2.2-fold down) were selected based on fold change and consistency in expression in an independent cohort. Thus, we derived a seven-gene signature, which included the five upregulated genes *FCGR1B, ANKRD22, CARD17, IFITM3,* and *TNFAIP6* and two downregulated genes *FCGBP* and *KLF12,* as a diagnostic biomarker for discrimination of active and latent tuberculosis infection. 

The short-listed genes were validated in an independent cohort, through bioinformatics analysis, which showed a statistically significant difference in gene expression between ATB and LTBI samples (Figure 3). This seven-gene signature has not been reported so far in discriminating active and latent tuberculosis. *FCGR1B, ANKRD22, CARD17, IFITM3,* and *TNFAIP6* genes are present in the 171 differentially expressed transcripts reported by Kaforou et al., but except for *FCGR1B*, none of the other genes are included in TB signatures classified by Kaforou et al. [14]. *FCGR1B* and *ANKRD22* are present in Zak-16 signature, which was applied in predicting the progression of LTBI into active TB [18]. The accuracy and performance of each gene of the seven-gene signature was calculated by receiver operating characteristic curve (ROC)-area under the curve (AUC).

The derived seven-gene signature has a sensitivity of 80–100% and specificity of 80–95%. Area under the curve (AUC) value of the genes ranged from 0.84 to 1. The ROC curves of seven-gene signature are illustrated in Figure 4, and sensitivity, specificity and AUC of seven-gene signature are presented in Table 5. A 27-transcript signature by Kaforou et al. [14] distinguished TB from LTBI, with sensitivity of 95% (95% CI 87–100) and specificity of 90% (95% CI 80–97). Gliddon et al. derived a three-transcript signature (*FCGR1A, ZNF296*, and *C1QB*) that differentiated TB from LTBI, with CI 95% (93.3–100%) [53]. The derived seven-gene expression signature is a promising biomarker, with high diagnostic accuracy in discrimination of active and latent TB infection.

## 5. Conclusions

The present study provides insight into differentially expressed host genes in active TB compared to latent TB infection among an African population that has both a high incidence (Cape Town) and a low incidence (Northern Malawi) of tuberculosis. The results of the functional annotation and pathway enrichment analysis led to identification of the primary pathways involving the upregulated genes (interferon and immune related) and downregulated genes (Wnt signaling and B cell signaling). 

We derived a seven-gene signature and validated the same in an independent cohort that can effectively discriminate between active and latent tuberculosis infection. Thus, the seven-gene signature could be further explored in other ethnic population for its potential in discrimination of active and latent tuberculosis infection.

## Figures and Tables

**Table 1:** (**A**) Top 10 upregulated genes in ATB vs. LTBI. (**B**) Top 10 downregulated genes in ATB vs. LTBI.

**(A)**				
**S.No**	**Gene**	**Probset ID**	**Mean ± SD Fold Change**	***p*-Value**
1	*FCGR1A*	ILMN_2176063	8.20 ± 1.14	1 × 10^−7^
2	*FCGR1B*	ILMN_2391051	9.80 ± 2.80	1 × 10^−7^
3	*FCGR1C*	ILMN_3247506	6.11 ± 2.24	1 × 10^−7^
4	*BATF2*	ILMN_1690241	12.4 ± 2.39	1 × 10^−7^
5	*GBP1*	ILMN_2148785	3.63 ± 0.32	1 × 10^−7^
6	*ANKRD22*	ILMN_1799848	10.25 ± 3.34	1 × 10^−7^
7	*GBP5*	ILMN_2114568	3.53 ± 1.03	1 × 10^−7^
8	*AIM2*	ILMN_1681301	4.10 ± 0.22	1 × 10^−7^
9	*GBP6*	ILMN_1756953	7.43 ± 2.82	1 × 10^−7^
10	*CASP4*	ILMN_1778059	4.40 ± 1.89	1 × 10^−7^
**(B)**				
**S.No**	**Gene**	**Probset ID**	**Mean ± SD Fold Change**	***p*-Value**
1	*NDRG2*	ILMN_2361603	2.17 ± 0.00	1 × 10^−7^
2	*KLF12*	ILMN_1762801	2.77 ± 0.00	1.4 × 10^−6^
3	*ANO9*	ILMN_1798679	4.05 ± 0.68	1 × 10^−7^
4	*CD79A*	ILMN_1659227	3.80 ± 0.50	1 × 10^−7^
5	*FCGBP*	ILMN_2302757	3.64 ± 0.28	1 × 10^−7^
6	*GZMK*	ILMN_1710734	2.22 ± 0.13	1 × 10^−7^
7	*CXCR3*	ILMN_1797975	2.27 ± 0.07	1 × 10^−7^
8	*MIEF2*	ILMN_1815923	2.06 ± 0.44	4.2 × 10^−6^
9	*CXCR5*	ILMN_2337928	2.86 ± 0.23	1 × 10^−7^
10	*CD27*	ILMN_1688959	2.15 ± 0.32	1 × 10^−7^

**Table 2:** Gene ontology and pathway enrichment analysis of upregulated genes.

S.No	Term/Pathway	*p* Value	Genes
GENE ONTOLOGY			
1	GO:0045087 ~ innate immune response	1.6 × 10^−10^	*CLEC4D, JAK2, NAIP, AIM2, CASP4, CAMP, C1QB, C1QC, CR1, DEFA1B, DEFA3, DEFA4, IL27, SLPI, SERPING1,* and *TLR2*
2	GO:0006955 ~ immune response	1 × 10^−6^	*CD274, FCAR, FCGR2C, FCGR1A, FCGR1B, AIM2, CEACAM,8, C1QC, DEFA1B, GBP6, SLPI,* and *TLR2*
3	GO:0050900 ~ leukocyte migration	1 × 10^−5^	*CD177, CEACAM1, CEACAM6, CEACAM8, GAS6, ITGB3,* and *SELP*
4	GO:0019731 ~ antibacterial humoral response	3 × 10^−5^	*CAMP, DEFA1B, DEFA3, DEFA4,* and *SLPI*
5	GO:0042742 ~ defense response to bacterium	3.2 × 10^−4^	*CLEC4D, ANXA3, CAMP, DEFA3, GBP6,* and *HP*
6	GO:0050830 ~ defense response to Gram-positive bacterium	4.1 × 10^−4^	*CAMP, DEFA1B, DEFA3, DEFA4,* and *TLR2*
7	GO:0002576 ~ platelet degranulation	8.5 × 10^−4^	*GAS6, ITGB3, MMRN1, SELP,* and *SERPING1*
8	GO:0006954 ~ inflammatory response	9.1 × 10^−4^	*NAIP, TNFAIP6, AIM2, CASP4, GBP5, IL27, SELP,* and *TLR2*
9	GO:0031640 ~ killing of cells of other organism	0.0014	*DEFA1B, DEFA3,* and *DEFA4*
10	GO:0042981 ~ regulation of apoptotic process	0.0018	*JAK2, CASP4, CASP5, CARD16, CARD17,* and *OLFM4*
KEGG PATHWAY			
1	has05150:Staphylococcus aureus infection	3 × 10^−6^	*FCAR, FCGR2C, FCGR1A, C1QB, C1QC,* and *SELP*
2	has05140:Leishmaniasis	1 × 10^−5^	*FCGR2C, FCGR1A, JAK2, CR1, MAPK14,* and *TLR2*
3	has05152:Tuberculosis	9.4 × 10^−5^	*FCGR2C, FCGR1A, CAMP, CR1, MAPK14,* and *TLR2*
4	has05322:Systemic lupus erythematosus	0.0025	*FCGR1A, C1QB, C1QC, HIST1H4H,* and *HIST2H2AB*
5	has04610:Complement and coagulation cascades	0.0032	*C1QB, C1QC, CR1,* and *SERPING1*
6	has04145:Phagosome	0.0038	*FCAR, FCGR2C, FCGR1A, ITGB3,* and *TLR2*
7	has05133:Pertussis	0.0040	*C1QB, C1QC, MAPK14,* and *SERPING1*
REACTOME PATHWAY			
1	Neutrophil degranulation	2 × 10^−14^	*CR1, TNFAIP6, DEFA4, MGST1, HP,* *MCEMP1, GPR84, OLFM4, MAPK14,* *FCAR, SELP, CEACAM1, CLEC4D, SLPI,* *CEACAM6, DEFA1B, CEACAM8, FCGR2C,* *CAMP, CD177, TLR2,* and *SIGLEC5*
2	Immune System	5.3 × 10^−12^	*C1QB, CD274, TNFAIP6, ITGB3, MGST1,* *HPIL27, GPR84, IFIT3, FCAR, CASP4, DEFA1B* *JAK2, FCGR1A, GBP1, FCGR1B, CAMP, CD177* *GBP6, GBP5, CR1, RSAD2, DEFA4, DEFA3,* *NRG1, MCEMP1, OLFM4, MAPK14, SELP,* *CEACAM1, CLEC4D, AIM2, SLPI, CEACAM6,* *SERPING1, CEACAM8, FCGR2C, TLR2,* *SIGLEC5,* and *C1QC*
3	Innate Immune System	6.8 × 10^−10^	*C1QB, TNFAIP6, MGST1, HP, GPR84, FCAR,* *CASP4, DEFA1B, FCGR1A, CAMP, CD177,* *CR1, DEFA4, DEFA3, MCEMP1, OLFM4,* *MAPK14, SELP, CEACAM1, CLEC4D, AIM2,* *SLPI, CEACAM6, SERPING1, CEACAM8,* *FCGR2C, TLR2, SIGLEC5,* and *C1QC*
4	Interferon Signaling	8.8 × 10^−8^	*GBP6, GBP5, RSAD2, JAK2, FCGR1A, GBP1,* *FCGR1B,* and *IFIT3*
5	Interferon γ signaling	1.4 × 10^−6^	*GBP6, GBP5, FCGR1A, JAK2, GBP1,* and *FCGR1B*
6	Fibronectin matrix formation	1.7 × 10^−5^	*CEACAM1, CEACAM6,* and *CEACAM8*
7	RMTs methylate histone arginines	3.7 × 10^−5^	*SMARCD3, HIST2H2AB, HIST1H4H,* and *JAK2*
8	α-defensins	6.7 × 10^−5^	*DEFA4, DEFA3,* and *DEFA1B*
9	Cell surface interactions at the vascular wall	7.5 × 10^−5^	*SELP, CEACAM1, CEACAM6, ITGB3,* *CEACAM8, GAS6,* and *CD177*
10	Cytokine signaling in immune system	3 × 10^−4^	*GBP6, GBP5, RSAD2, ITGB3, IL27, NRG1,* *JAK2, FCGR1A, MAPK14, GBP1, FCGR1B,* and *IFIT3*

**Table 3:** Gene ontology and pathway enrichment analysis of downregulated genes.

Scheme	Term/Pathway	*p* Value	Genes
GENE ONTOLOGY			
1	GO:0016055 ~ Wnt signaling pathway	0.0012	*NDRG2, WNT7A, CSNK1E, DKK3, TCF7,* and *TLE2*
2	GO:0030154 ~ cell differentiation	0.0035	*BLK, FCRLA, NDRG2, SFMBT1, SPIB, FLNB, LAMA5,* and *MATK*
3	GO:0006954 ~ inflammatory response	0.0056	*CXCR3, CD27, GPR68, TNFRSF25, IL23A, NCR3,* and *NFATC3*
4	GO:0042127 ~ regulation of cell proliferation	0.0081	*BLK, CD27, TNFRSF25, LAMA5, TCF7*
5	GO:0006955 ~ immune response	0.0093	*CXCR5, CD27, CD8B, TNFRSF25, NCR3, VPREB3,* and *TCF7*
6	GO:0050853 ~ B cell receptor signaling pathway	0.022	*BLK, CD19,* and *CD79A*
7	GO:0007275 ~ multicellular organism development	0.024	*TCL1A, TNFRSF25, DKK3, EBF1*, *ID3, PLXNA3,* and *PAQR7*
KEGG PATHWAY			
1	has05340: primary immunodeficiency	4.9 × 10^−4^	*CD19, CD79A, CD8B,* and *TNFRSF13C*
2	hsa04060: cytokine-cytokine receptor interaction	7.6 × 10^−4^	*CXCR3, CXCR5, CD27, TNFRSF13C, TNFRSF25, IL11RA,* and *IL23A*
3	hsa04640: hematopoietic cell lineage	0.0074	*CD19, CD8B, IL11RA,* and *MS4A1*
4	has04360: Axon guidance	0.020	*EPHA4, EPHB6, NFATC3,* and *PLXNA3*
5	hsa04310: Wnt signaling pathway	0.025	*WNT7A, CSNK1E, NFATC3,* and *TCF7*
6	hsa04662: B cell receptor signaling pathway	0.040	*CD19, CD79A,* and *NFATC3*
REACTOME PATHWAY			
1	Repression of WNT target genes	0.0062	*TLE2, TCF7*
2	Loss of proteins required for interphase microtubule organization from the centrosome	0.0153	*HAUS5, CSNK1E,* and *SFI1*
3	Loss of Nlp from mitotic centrosomes	0.0153	*HAUS5, CSNK1E,* and *SFI1*
4	AURKA Activation by TPX2	0.0170	*HAUS5, CSNK1E,* and *SFI1*
5	TNFs bind their physiological receptors	0.0204	*CD27, TNFRSF25*
6	Recruitment of mitotic centrosome proteins and complexes	0.0216	*HAUS5, CSNK1E,* and *SFI1*
7	Centrosome maturation	0.0230	*HAUS5, CSNK1E,* and *SFI1*
8	Regulation of PLK1 Activity at G2/M Transition	0.0299	*HAUS5, CSNK1E,* and *SFI1*
9	Recruitment of NuMA to mitotic centrosomes	0.0342	*HAUS5, CSNK1E,* and *SFI1*
10	Antigen activates B Cell Receptor (BCR) leading to generation of second messengers	0.0397	*BLK, CD79A,* and *CD19*

**Table 4:** (**A**) Closely interlinked regions in the PPI network are clustered by MCODE analysis. (**B**) Top-ranked hub nodes are categorized based on MCC Method.

**(A)**				
**Custer**	**MCODE Score (Density X No. of Nodes)**	**Nodes**	**Edges**	**Node IDs**
1	7	7	21	*CLEC4D, GPR84, CEACAM8,* and *CEACAM1* *CD177, FCAR,* and *MCEMP1*
2	5	13	30	*CASP4, DEFA4, CAMP, FCGR1A, TLR2, CASP5, CR1, AIM2, SLPI,* and *NAIP* *OLFM4, FCGR2A,* and *HP*
**(B)**				
**Rank**	**Hub Nodes**			**MCC Score**
1	*CEACAM8*			770
2	*FCAR*			750
3	*CLEC4D*			730
4	*CD177*			723
5	*CEACAM1*			722
6	*MCEMP1*			720
7	*GPR84*			720
8	*TLR2*			76
9	*FCGR1A*			70
10	*CAMP*			62
11	*FCGR2A*			52
12	*OLFM4*			50
13	*DEFA4*			48
14	*HP*			43
15	*CASP4*			38
16	*CASP5*			38
17	*AIM2*			33
18	*CR1*			31
19	*SLPI*			30
20	*NAIP*			24

**Table 5:** Sensitivity, specificity, and AUC of seven-gene signature in discriminating active and latent tuberculosis.

S.No.	Gene	Sensitivity (95% CI)	Specificity (95% CI)	AUC	95% CI
1	*FCGR1B*	100%	95%	1	1.000 to 1.000
2	*ANKRD22*	90%	85%	0.94	0.8689 to 1.000
3	*CARD17*	85%	90%	0.96	0.9152 to 1.000
4	*IFITM3*	85%	85%	0.85	0.7261 to 0.9789
5	*TNFAIP6*	80%	90%	0.89	0.7831 to 1.000
6	*KLF12*	75%	80%	0.84	0.7222 to 0.9678
7	*FCGBP*	80%	80%	0.9	0.8103 to 0.9947

AUC: Area under the ROC curve; CI: Confidence interval.

## Data Availability

Gene expression dataset GSE37250 can be accessed at https://www.ncbi.nlm.nih.gov/geo/query/acc.cgi?acc=GSE37250 (accessed date 15 July 2021), and validation data GSE19491 can be accessed at https://www.ncbi.nlm.nih.gov/geo/query/acc.cgi?acc=GSE19491 (accessed on 19 April 2021). Both these data can be accessed from Gene Expression Omnibus (GEO) database.

## Supplementary Materials

The following supporting information can be downloaded at: https://www.mdpi.com/article/10.3390/genes13040616/s1, Table S1. List of most upregulated genes and downregulated genes in ATB vs. LTBI. Figure S1. Heatmap of upregulated genes in ATB vs. LTBI. Figure S2. Heatmap of downregulated genes in ATB vs. LTBI.
