# Supplementary material for: Transcriptional Profiling and Deriving a Seven-Gene Signature That Discriminates Active and Latent Tuberculosis: An Integrative Bioinformatics Approach
Source: Genes (Basel). 2022 Mar 29;13(4):616. doi: 10.3390/genes13040616 (PMC9032611; doi:10.3390/genes13040616)
Supplement: Supplementary file 1 [file genes-13-00616-s001.zip › genes-1579823-Supplementary-Figures.pdf]

Supplementary Figures

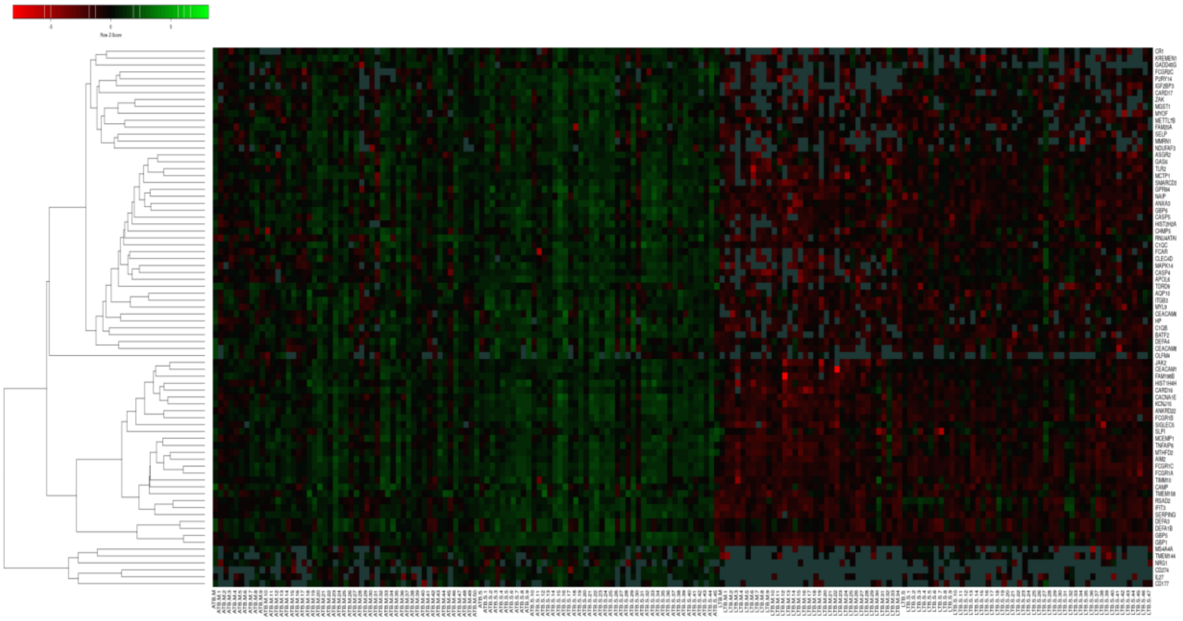

Figure S1. Results of differential gene expression pattern between ATB and LTBI. the expression levels are scaled from dark red (low expression) to dark green (high expression).

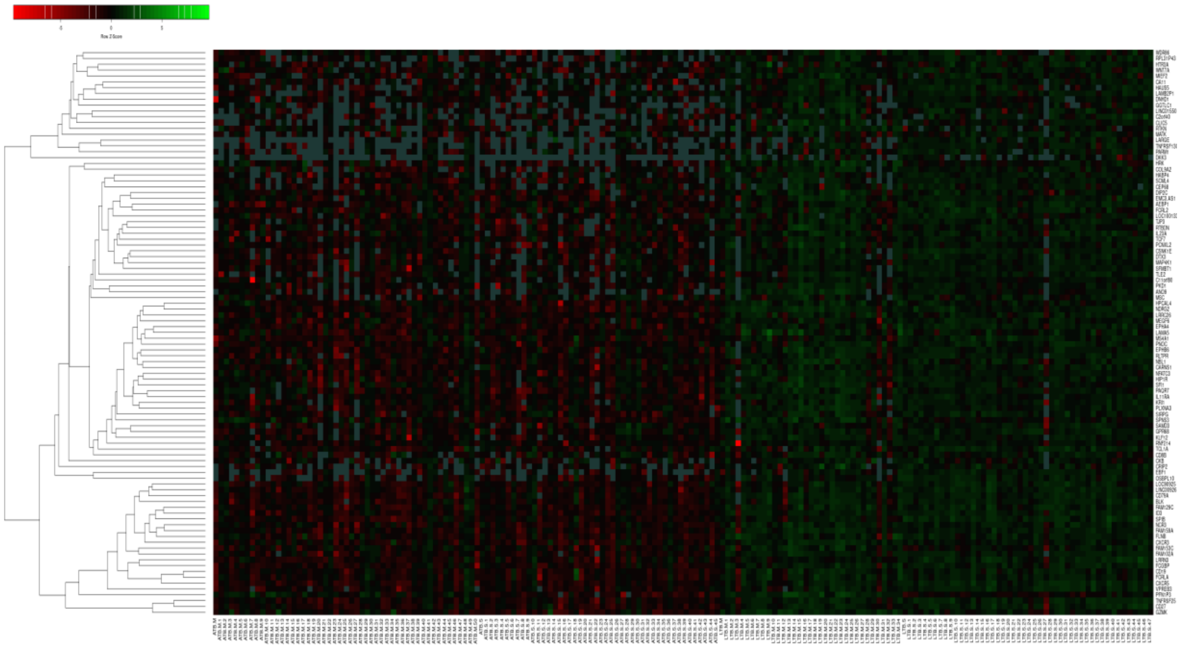

Figure S2. Heatmap of downregulated genes in ATB vs LTBI. The expression levels are scaled from dark red (low expression) to dark green (high expression).
