# Supplementary material for: Transcriptional Profiling and Deriving a Seven-Gene Signature That Discriminates Active and Latent Tuberculosis: An Integrative Bioinformatics Approach
Source: Genes (Basel). 2022 Mar 29;13(4):616. doi: 10.3390/genes13040616 (PMC9032611; doi:10.3390/genes13040616)
Supplement: Supplementary file 1 [file genes-13-00616-s001.zip › genes-1579823-Supplementary-Table.pdf]

Supplementary Table S1

ATB vs LTB up regulated genes common to South Africa & Malawi.

S.no	Gene	Probset ID	Mean fold change	Mean P-value
1.	*FCGR1A*	ILMN_2176063	8.20	1E-07
2.	*FCGR1B*	ILMN_2391051	9.80	1E-07
3.	*FCGR1C*	ILMN_3247506	6.11	1E-07
4.	*BATF2*	ILMN_1690241	12.4	1E-07
5.	*GBP1*	ILMN_2148785	3.63	1E-07
6.	*ANKRD22*	ILMN_1799848	10.25	1E-07
7.	*GBP5*	ILMN_2114568	3.53	1E-07
8.	*AIM2*	ILMN_1681301	4.10	1E-07
9.	*GBP6*	ILMN_1756953	7.43	1E-07
10.	*CASP4*	ILMN_1778059	4.40	1E-07
11.	*SMARCD3*	ILMN_2408987	4.43	1E-07
12.	*TNFAIP6*	ILMN_1785732	3.47	1E-07
13.	*C1QB*	ILMN_1796409	11.69	1E-07
14.	*SERPING1*	ILMN_1670305	8.10	1E-07
15.	*CARD16*	ILMN_1726591	3.91	1E-07
16.	*KCNJ15*	ILMN_2396903	3.45	1E-07
17.	*FAM198B*	ILMN_1672124	3.48	1E-07
18.	*ANXA3*	ILMN_1694548	4.40	1E-07
19.	*MTHFD2*	ILMN_2405521	3.01	1E-07
20.	*CACNA1E*	ILMN_1664047	5.80	1E-07
21.	*CEACAM1*	ILMN_1664330	3.18	1E-07
22.	*TDRD9*	ILMN_2169490	4.11	1.5E-07
23.	*CARD17*	ILMN_3238525	6.23	1E-07
24.	*MCTP1*	ILMN_1654685	3.53	1E-07
25.	*TMEM158*	ILMN_1792455	2.98	1E-07
26.	*TLR2*	ILMN_1772387	3.50	1E-07
27.	*MMRN1*	ILMN_1660114	4.52	1E-07
28.	*IGF2BP3*	ILMN_1807423	4.52	1E-07
29.	*KREMEN1*	ILMN_1772697	6.51	1E-07
30.	*HIST2H2AB*	ILMN_1747589	4.63	1E-07
31.	*MGST1*	ILMN_1781952	3.12	1E-07
32.	*NRG1*	ILMN_1737252	3.37	7E-07
33.	*CASP5*	ILMN_1722158	5.81	1E-07
34.	*JAK2*	ILMN_1683178	3.12	1E-07
35.	*GAS6*	ILMN_1784749	3.35	1E-07
36.	*CHMP5*	ILMN_2094166	3.45	1E-07
37.	*FAM20A*	ILMN_1812091	4.27	1E-07
38.	*MCEMP1*	ILMN_1762713	3.87	1E-07
39.	*P2RY14*	ILMN_1764036	5.28	1E-07
40.	*MAPK14*	ILMN_2388090	3.84	1E-07
41.	*C1QC*	ILMN_1785902	5.89	1E-07
42.	*IFIT3*	ILMN_1664543	3.58	1E-07
43.	*SELP*	ILMN_1715417	4.09	1E-07
44.	*TMEM144*	ILMN_1708110	3.68	8.5E-07
45.	*HIST1H4H*	ILMN_1751120	3.53	1E-07
46.	*NAIP*	ILMN_1760189	3.91	1E-07
47.	*ASGR2*	ILMN_1694966	3.70	1E-07
48.	*APOL6*	ILMN_1687201	3.29	1E-07
49.	*CAMP*	ILMN_1688580	3.05	1E-07
50.	*CLEC4D*	ILMN_1808979	3.32	1.05E-06
51.	*METTL7B*	ILMN_2064725	4.70	1E-07
52.	*FCAR*	ILMN_2365091	3.59	1E-07
53.	*HP*	ILMN_1812433	4.77	1E-07
54.	*MS4A4A*	ILMN_2370336	3.53	6E-07

55.	*GPR84*	ILMN_1785345	3.16	1E-07
56.	*MYL9*	ILMN_1776953	5.67	1E-07
57.	*ITGB3*	ILMN_1733324	3.38	1E-07
58.	*CR1*	ILMN_1767193	3.48	1E-07
59.	*RSAD2*	ILMN_1657871	3.27	1E-06
60.	*CEACAM8*	ILMN_1806056	5.14	3E-07
61.	*FCGR2C*	ILMN_2331121	3.39	1E-07
62.	*RNU4ATAC*	ILMN_3240594	3.34	6.5E-07
63.	*ZAK*	ILMN_1698803	3.39	1E-07
64.	*CEACAM6*	ILMN_1712522	3.82	3.5E-07
65.	*CD177*	ILMN_3251610	4.94	2E-07
66.	*NDUFAF3*	ILMN_1702120	3.89	1E-07
67.	*SLPI*	ILMN_2114720	3.49	3E-07
68.	*TIMM10*	ILMN_1765332	3.02	2E-07
69.	*GADD45G*	ILMN_1651498	3.10	2.5E-07
70.	*OLFM4*	ILMN_2116877	5.23	4.5E-06
71.	*SIGLEC5*	ILMN_1740298	3.25	4.5E-07
72.	*DEFA4*	ILMN_1753347	4.54	6.5E-07
73.	*IL27*	ILMN_1753758	4.73	8.5E-07
74.	*MYOF*	ILMN_2370976	4.19	7.7E-06
75.	*AQP10*	ILMN_2090004	3.33	3.5E-06
76.	*CD274*	ILMN_1701914	4.77	1.2E-05
77.	*DEFA1B*	ILMN_2102721	3.45	2.3E-05
78.	*DEFA3*	ILMN_2165289	3.23	2.6E-05
79.	*LHFPL2*	ILMN_1747744	2.855	1.00E-07
80.	*LOC728744*	ILMN_1654389	7.795	1.00E-07
81.	*PSTPIP2*	ILMN_1713058	2.49	1.00E-07
82.	*TLR5*	ILMN_1722981	2.54	1.00E-07
83.	*DTX3L*	ILMN_1784380	2.275	1.65E-06
84.	*GK*	ILMN_2393296	2.655	1.00E-07
85.	*LAP3P2*	ILMN_3295494	2.855	1.00E-07
86.	*LOC440731*	ILMN_1683250	2.48	1.00E-07
87.	*PARP9*	ILMN_2053527	2.155	1.00E-07
88.	*GRAMD1B*	ILMN_3237376	3.285	1.22E-05
89.	*HIST1H3D*	ILMN_1721127	2.635	1.00E-07
90.	*ATF3*	ILMN_2374865	2.605	1.68E-05
91.	*ODF3B*	ILMN_2209614	2.03	1.24E-05
92.	*HIST2H2AA3*	ILMN_1659047	2.275	1.00E-07
93.	*LMNB1*	ILMN_2126706	2.6	1.00E-07
94.	*SLC26A8*	ILMN_1755843	3.845	1.00E-07
95.	*TIFA*	ILMN_1686454	2.655	1.47E-04
96.	*APOL1*	ILMN_1761793	2.92	4.69E-05
97.	*HIST2H2AC*	ILMN_1768973	2.48	1.00E-07
98.	*HIST2H2AA4*	ILMN_3242900	2.245	1.00E-07
99.	*HIST1H2BD*	ILMN_1758623	2.475	5.50E-07
100.	*P2RY13*	ILMN_1664525	2.57	1.00E-07
101.	*LAP3*	ILMN_1683792	2.475	1.00E-07
102.	*C5*	ILMN_1746819	2.24	1.00E-07
103.	*ADCY3*	ILMN_1676893	2.49	1.00E-07
104.	*WDFY3*	ILMN_1697493	2.075	7.00E-07
105.	*NLRC4*	ILMN_1796976	2.64	1.00E-07
106.	*LOC648984*	ILMN_1801254	2.59	1.00E-07
107.	*LOC100130828*	ILMN_3256712	2.63	1.00E-07
108.	*TRIM22*	ILMN_1779252	2.165	1.00E-07
109.	*IFITM3*	ILMN_1805750	2.715	1.00E-07
110.	*RTP4*	ILMN_2173975	2.675	5.20E-06
111.	*HIST1H4D*	ILMN_1676580	2.9	3.42E-05
112.	*LOC648710*	ILMN_1710698	2.125	4.50E-06

113.	*S100A8*	ILMN_1729801	2.36	1.00E-07
114.	*HPSE*	ILMN_1779547	2.005	1.00E-07
115.	*PLSCR1*	ILMN_1745242	2.63	1.00E-07
116.	*C1QA*	ILMN_1737918	2.435	1.00E-07
117.	*C2*	ILMN_1710740	2.94	1.00E-07
118.	*LOC653610*	ILMN_1695435	2.72	1.00E-07
119.	*GALNT14*	ILMN_1722524	3.615	5.00E-07
120.	*TCN2*	ILMN_1740572	2.37	1.00E-07
121.	*CD36*	ILMN_1784863	2.795	1.00E-07
122.	*LOC388572*	ILMN_3294156	3.065	3.77E-05
123.	*HCAR2*	ILMN_1750497	2.58	3.00E-07
124.	*EPSTI1*	ILMN_2388547	2.62	1.00E-07
125.	*LRG1*	ILMN_1805228	2.08	2.00E-07
126.	*LOC651738*	ILMN_1674789	2.135	1.57E-05
127.	*CDK5RAP2*	ILMN_2415529	2.385	1.50E-04
128.	*ADM*	ILMN_1708934	2.235	1.00E-07
129.	*FAM26F*	ILMN_2066849	2.78	1.00E-07
130.	*CREB5*	ILMN_1728677	2.175	1.00E-07
131.	*LIMK2*	ILMN_2367671	2.565	1.00E-07
132.	*NOD2*	ILMN_1762594	1.945	2.00E-06
133.	*DDX60L*	ILMN_3243928	2.425	1.00E-07
134.	*GCH1*	ILMN_1812759	1.99	1.00E-07
135.	*LOC100170939*	ILMN_3238814	2.5	1.00E-07
136.	*LOC644615*	ILMN_1784005	2.37	5.10E-06
137.	*MIR21*	ILMN_3310840	2.63	1.00E-07
138.	*CCRL2*	ILMN_3190833	2.47	5.00E-07
139.	*LOC728519*	ILMN_1679620	2.63	1.00E-07
140.	*VNN1*	ILMN_1674574	2.695	1.00E-07
141.	*LOC100132287*	ILMN_3234783	2.175	0.0001902
142.	*SLAMF8*	ILMN_1667224	2.255	0.0003901
143.	*IL15*	ILMN_2273053	2.15	0.0000241
144.	*TXN*	ILMN_1680314	2.02	1.00E-07
145.	*SLC22A4*	ILMN_2050911	2.09	1.00E-07
146.	*BMX*	ILMN_1796138	2.2	1.21E-05
147.	*DHRS9*	ILMN_1733998	2.285	1.00E-07
148.	*DSC2*	ILMN_1663119	2.69	1.00E-07
149.	*GPR141*	ILMN_2092333	2.465	1.00E-07
150.	*ZDHHC19*	ILMN_1766896	2.8	1.63E-05
151.	*HCAR3*	ILMN_1677693	2.71	1.00E-07
152.	*KREMEN2*	ILMN_1761321	2.135	2.40E-06
153.	*IL15RA*	ILMN_1665682	2.16	1.10E-04
154.	*RTN2*	ILMN_1749115	1.935	1.00E-07
155.	*LOC728093*	ILMN_3231554	5.095	8.00E-07
156.	*RAB20*	ILMN_1708881	2.5	1.23E-05
157.	*NAMPT*	ILMN_1753111	2.505	7.00E-07
158.	*ANKRD9*	ILMN_2048607	2.11	1.82E-04
159.	*FZD2*	ILMN_1653711	2.06	7.70E-06
160.	*ITGA2B*	ILMN_1721888	2.825	3.07E-05
161.	*SLC6A12*	ILMN_1780831	2.97	1.00E-07
162.	*PRRG4*	ILMN_1661809	2.685	1.00E-07
163.	*DDIAS*	ILMN_1790100	2.09	1.00E-07
164.	*SUCNR1*	ILMN_1681601	2.23	4.50E-06
165.	*NTNG2*	ILMN_1806448	2.695	2.92E-05
166.	*MARCO*	ILMN_1731503	2.16	3.00E-07
167.	*SAMD4A*	ILMN_2119297	1.91	1.67E-05
168.	*HIST1H4E*	ILMN_1681542	2.535	2.10E-06
169.	*LILRA5*	ILMN_1726545	2.045	1.00E-07
170.	*SLITRK4*	ILMN_2199768	2.55	1.00E-07

171.	*DNAJC25-GNG10*	ILMN_1767809	2.64	1.00E-07
172.	*ALPL*	ILMN_1701603	1.925	3.59E-05
173.	*AP5B1*	ILMN_1717594	1.95	2.81E-05
174.	*TCN1*	ILMN_1768469	2.625	5.00E-07
175.	*DKFZP434B2016*	ILMN_1752798	1.935	1.40E-06
176.	*GNG10*	ILMN_1757074	2	1.40E-06
177.	*MPO*	ILMN_1705183	2.32	3.30E-06
178.	*PPP1R3B*	ILMN_1712236	2.225	7.00E-07
179.	*CCR2*	ILMN_2276996	2.135	1.00E-07
180.	*FCGR2A*	ILMN_1706523	1.755	1.22E-05
181.	*LOC401281*	ILMN_1797409	2.2	5.00E-04
182.	*TREML1*	ILMN_1690783	2.33	3.30E-06
183.	*LOC645822*	ILMN_3216298	2.015	5.91E-05
184.	*FPR2*	ILMN_2392569	2.075	7.00E-07
185.	*INSL3*	ILMN_2226271	2.425	4.99E-04
186.	*LOC388444*	ILMN_3237428	2.545	4.15E-04
187.	*COL17A1*	ILMN_1651282	2.205	3.24E-04
188.	*GLRXP3*	ILMN_3280402	2.43	1.00E-07
189.	*SLC2A14*	ILMN_1668865	2.535	1.00E-07
190.	*CSTA*	ILMN_1669888	2.91	1.00E-07
191.	*LOC643313*	ILMN_1789965	1.82	5.87E-05
192.	*ST3GAL4*	ILMN_2204545	1.82	1.50E-04
193.	*SOD2*	ILMN_2406501	1.79	3.57E-05
194.	*LOC100134660*	ILMN_3247661	1.955	1.37E-05
195.	*NBN*	ILMN_2358041	2.195	1.50E-06
196.	*TMEM167A*	ILMN_3251560	2.465	1.00E-07
197.	*LY96*	ILMN_1724533	2.36	1.00E-07
198.	*TFPI*	ILMN_1662619	2.505	5.30E-06
199.	*SIPA1L2*	ILMN_1732923	2.415	1.00E-07
200.	*HIST1H2BJ*	ILMN_1658702	1.91	0.0003385
201.	*CEACAM3*	ILMN_1743570	2.145	0.0000015
202.	*SPHK1*	ILMN_2357134	2.835	1.00E-07
203.	*MT1G*	ILMN_1715401	2.285	4.00E-06
204.	*PLOD2*	ILMN_2410924	1.825	1.75E-04
205.	*LOC729915*	ILMN_3242786	3.195	4.57E-05
206.	*LOC100134822*	ILMN_3239734	2.055	2.96E-05
207.	*ABLIM3*	ILMN_1656940	3.165	1.00E-07
208.	*HIST1H2BC*	ILMN_1680937	1.925	1.00E-07
209.	*TMEM51*	ILMN_1674985	2.01	0.0003614
210.	*AQP12A*	ILMN_1757036	2.305	0.0000009
211.	*HIST1H2BE*	ILMN_1687947	2.85	1.00E-07
212.	*CA4*	ILMN_1695157	1.88	8.87E-05
213.	*KIAA1881*	ILMN_1852022	1.915	1.48E-04
214.	*MB21D1*	ILMN_1706645	2.565	8.40E-06
215.	*PPAP2C*	ILMN_1675523	1.765	4.72E-04
216.	*ELANE*	ILMN_1706635	2.725	6.67E-05
217.	*MAZ*	ILMN_1677997	2.375	1.00E-07
218.	*ASAP2*	ILMN_3194508	2.055	1.00E-07
219.	*LOC642267*	ILMN_1698519	1.845	9.30E-06
220.	*LOC100134728*	ILMN_3240900	2.01	1.00E-07
221.	*LOC730953*	ILMN_1658697	1.83	0.0002597
222.	*PSG9*	ILMN_1801776	2.51	0.0000015
223.	*TNFSF13*	ILMN_1784264	1.945	0.0001423
224.	*SLC2A3P2*	ILMN_1806015	1.915	0.0000076
225.	*CASP7*	ILMN_2373763	2.145	0.0000946
226.	*BCL6*	ILMN_1746053	2.29	0.0000089
227.	*LIN7A*	ILMN_1806293	3.01	1.00E-07
228.	*FAM160B1*	ILMN_3182275	2.34	1.00E-07

229.	*BEND7*	ILMN_3230880	1.92	0.0003788
230.	*HIST2H3D*	ILMN_3249188	2.435	0.0000066
231.	*ECM1*	ILMN_2329735	1.8	0.0000003
232.	*F5*	ILMN_1709233	1.775	0.0000023
233.	*LCN2*	ILMN_1692223	2.365	0.0000099
234.	*CDH6*	ILMN_1766675	2.355	0.0000081
235.	*TRIM6*	ILMN_1656910	2.62	0.000023
236.	*C3AR1*	ILMN_1787529	1.78	0.0000212
237.	*DEFA1*	ILMN_2193213	2.725	0.0000808
238.	*TSPAN9*	ILMN_1729453	1.9	0.0001377
239.	*TRPM2*	ILMN_2352380	2.32	0.0001445
240.	*LOC653867*	ILMN_1678633	1.91	0.0000178
241.	*SLA*	ILMN_2291954	1.84	0.0003179
242.	*CETP*	ILMN_2098013	2.395	0.0000005
243.	*S100A12*	ILMN_1748915	2.88	1.00E-07
244.	*MCMBP*	ILMN_1761411	2.36	1.00E-07
245.	*OPLAH*	ILMN_1711030	2.945	1.00E-07
246.	*ITGB5*	ILMN_2311166	2.05	0.0003537
247.	*CXCL10*	ILMN_1791759	2.26	0.00001
248.	*IFI44*	ILMN_1760062	2.865	1.00E-07
249.	*DENND1A*	ILMN_1728073	2.115	7.83E-05
250.	*KAZN*	ILMN_1798458	2.19	1.70E-06
251.	*GNG11*	ILMN_1782419	1.93	1.00E-07
252.	*TMC4*	ILMN_1803219	2.615	8.00E-07
253.	*SAMD14*	ILMN_1760688	2.605	1.69E-04
254.	*BPI*	ILMN_1766736	2.965	4.40E-06
255.	*HIST1H2BG*	ILMN_1716195	2.665	5.40E-06
256.	*SMPDL3A*	ILMN_1796349	1.96	2.00E-07
257.	*B3GNT8*	ILMN_1741389	1.705	5.17E-04
258.	*F13A1*	ILMN_1717163	2.02	1.00E-07
259.	*GUCY1A3*	ILMN_2131177	1.675	2.76E-04
260.	*EGF*	ILMN_1690733	2.215	4.60E-06
261.	*STEAP4*	ILMN_1772036	2.295	1.00E-07
262.	*KRT75*	ILMN_1721247	2.09	1.86E-04
263.	*CLEC5A*	ILMN_1780465	2.89	9.81E-05
264.	*TGFB1I1*	ILMN_2389876	2.205	1.00E-04
265.	*CMTM5*	ILMN_1775373	1.89	2.65E-05
266.	*TECPR2*	ILMN_1764456	2.755	3.38E-05
267.	*PFKFB2*	ILMN_1723436	1.945	6.70E-05
268.	*IFI6*	ILMN_2347798	1.815	4.29E-05
269.	*GBP3*	ILMN_1725314	2.84	3.85E-06
270.	*LOC653600*	ILMN_1693262	3.59	1.77E-04
271.	*LOC648733*	ILMN_1668588	1.97	1.65E-05
272.	*LOC100130904*	ILMN_3185147	2.96	1.65E-06
273.	*CECR6*	ILMN_1702229	1.86	1.90E-06
274.	*LOC100132317*	ILMN_3241912	2.465	7.65E-06
275.	*MCTP2*	ILMN_1792682	2.28	2.50E-07
276.	*FYB*	ILMN_2280548	2.94	1.00E-07
277.	*FAM153B*	ILMN_1736819	2.21	9.00E-07
278.	*DDX60*	ILMN_1795181	1.85	2.00E-07
279.	*LOC100133077*	ILMN_3287106	2.35	4.20E-06
280.	*MEF2A*	ILMN_1661888	2.25	1.16E-05
281.	*MXI1*	ILMN_1794074	2.975	2.50E-07
282.	*C1GALT1C1*	ILMN_2401730	2.34	4.00E-07
283.	*PTPN20*	ILMN_1680945	2.13	4.57E-05
284.	*MMP9*	ILMN_1796316	2.405	3.50E-07
285.	*ERLIN1*	ILMN_1730731	2.015	2.76E-05
286.	*LOC642120*	ILMN_1659639	2.015	1.05E-05

287.	*CLEC1A*	ILMN_1691339	2.385	3.15E-06
288.	*RPS6KA3*	ILMN_1652736	1.35	2.05E-04
289.	*LOXL3*	ILMN_1733515	2.385	4.00E-07
290.	*PPBP*	ILMN_1767281	2.055	1.00E-07
291.	*ARG2*	ILMN_1800898	2.06	2.40E-06
292.	*BACH1*	ILMN_1807181	2.605	1.00E-07
293.	*ASPRV1*	ILMN_1762284	1.835	1.79E-04
294.	*LILRA6*	ILMN_1694243	2.07	8.95E-06
295.	*LOC100129904*	ILMN_3259736	2.03	6.94E-05
296.	*GPER1*	ILMN_1795298	2.93	1.70E-06
297.	*ADGRG3*	ILMN_1765941	1.665	1.20E-04
298.	*LOC650546*	ILMN_1814812	2.025	2.00E-06
299.	*IFI44L*	ILMN_1723912	2.76	2.06E-04
300.	*PF4V1*	ILMN_1745522	2.07	2.57E-05
301.	*GLT1D1*	ILMN_1656327	1.835	1.15E-04
302.	*NUDT16P1*	ILMN_1716468	2.25	1.43E-04
303.	*PNKD*	ILMN_1774604	1.78	5.29E-05
304.	*IFIT2*	ILMN_1739428	1.965	1.00E-07
305.	*ELF2*	ILMN_2252295	2.875	1.50E-06
306.	*SLC22A16*	ILMN_1763609	3.095	1.00E-07
307.	*YOD1*	ILMN_1678919	2.135	4.05E-06
308.	*NDUFV3*	ILMN_2387731	2.11	1.05E-05
309.	*XK*	ILMN_1759117	2.575	1.98E-04
310.	*MBOAT2*	ILMN_1777853	1.94	9.43E-05
311.	*CAV2*	ILMN_2360730	1.69	1.19E-04
312.	*ADORA2B*	ILMN_1703946	2.04	6.80E-06
313.	*LRP1*	ILMN_1669772	2.125	1.08E-04
314.	*EPHB1*	ILMN_1692261	1.175	2.76E-04
315.	*LSMEM1*	ILMN_1804895	2.365	5.30E-06
316.	*EVI2A*	ILMN_2369018	3.13	1.00E-07
317.	*SH3BGRL2*	ILMN_1762764	1.745	7.00E-06
318.	*ESAM*	ILMN_1668092	1.835	2.47E-04
319.	*FRMD4B*	ILMN_1700143	2.13	6.85E-06
320.	*LOC100131360*	ILMN_3215367	1.7	5.54E-05
321.	*C4orf32*	ILMN_2069821	1.975	4.29E-05
322.	*MANSC1*	ILMN_2142752	2.11	1.10E-05
323.	*UBE2C*	ILMN_2301083	1.88	1.53E-04
324.	*TPM1*	ILMN_2360710	1.7	1.20E-05
325.	*ATG3*	ILMN_2163732	1.77	1.57E-05
326.	*C9orf72*	ILMN_1666742	1.87	2.15E-05
327.	*SPTB*	ILMN_1782845	2.27	8.96E-05
328.	*LEPR*	ILMN_2234956	1.885	2.32E-05
329.	*PROK1*	ILMN_1716259	2.075	1.30E-05
330.	*PEAR1*	ILMN_2407669	2.17	1.36E-05
331.	*PGLYRP1*	ILMN_1704870	1.68	1.68E-04
332.	*LOC642103*	ILMN_1712999	1.725	1.71E-04
333.	*METTL9*	ILMN_2402629	2.725	1.00E-07
334.	*CALD1*	ILMN_1730487	2.5	1.00E-07
335.	*IFIT5*	ILMN_1696654	1.83	3.25E-05
336.	*HIST2H4B*	ILMN_3238233	1.64	1.30E-04
337.	*SERPINB8*	ILMN_2397028	2	2.60E-05
338.	*LGALSL*	ILMN_1673548	2.345	1.00E-07
339.	*FBLN1*	ILMN_1700541	2.72	2.76E-05
340.	*MS4A4E*	ILMN_3200211	1.81	4.12E-05
341.	*S1PR3*	ILMN_1703531	2.28	3.16E-05
342.	*HNMT*	ILMN_1705984	1.95	4.59E-05
343.	*LPGAT1*	ILMN_2151277	1.855	3.75E-05
344.	*UBFD1*	ILMN_1700811	1.89	5.60E-05

345.	*H3.Y*	ILMN_3278627	1.805	2.12E-04
346.	*MSI2*	ILMN_1713088	1.875	4.36E-05
347.	*ABCC4*	ILMN_2194009	2.33	1.00E-07
348.	*TMEM88*	ILMN_1757129	1.78	1.98E-04
349.	*CTNNAL1*	ILMN_2136446	2.01	4.68E-05
350.	*OLR1*	ILMN_1723035	2.64	5.83E-05
351.	*MIR219A2*	ILMN_3311015	1.66	1.21E-04
352.	*KIFC3*	ILMN_1710354	2.575	1.00E-07
353.	*ACVRL1*	ILMN_3250257	1.915	6.99E-05
354.	*TRIM9*	ILMN_1763433	2.085	7.09E-05
355.	*MGAM*	ILMN_1714643	1.66	1.31E-04
356.	*MS4A3*	ILMN_1695530	1.86	7.69E-05
357.	*HOXA9*	ILMN_1739582	1.835	1.40E-04
358.	*TRPM6*	ILMN_1814296	1.825	1.29E-04
359.	*SAMSN1*	ILMN_2171289	2.03	1.44E-04
360.	*ARL11*	ILMN_1808383	1.78	1.55E-04
361.	*LOC650261*	ILMN_1729635	1.79	1.63E-04
362.	*GRIK1*	ILMN_1651817	1.68	2.32E-04
363.	*HIST1H2AD*	ILMN_1742143	1.865	1.97E-04
364.	*FBXL13*	ILMN_1791253	1.725	2.12E-04
365.	*SYTL4*	ILMN_1719599	1.995	2.19E-04
366.	*REPS2*	ILMN_1724668	1.845	2.78E-04
367.	*BCAT1*	ILMN_1766169	2.62	1.00E-07
368.	*CLEC1B*	ILMN_1745103	3.925	1.00E-07
369.	*HEPACAM2*	ILMN_2388263	2.095	2.36E-04
370.	*TP53I3*	ILMN_2358919	2.03	2.49E-04
371.	*MRPL35*	ILMN_1753016	1.195	2.60E-04
372.	*ARG1*	ILMN_1812281	2.625	2.77E-04
373.	*ACSL3*	ILMN_1666096	1.76	3.29E-04
374.	*CLEC12A*	ILMN_2292178	1.995	3.02E-04
375.	*RHCE*	ILMN_2408663	2.14	7.02E-04
376.	*SPARC*	ILMN_1796734	1.79	3.81E-04
377.	*HERC5*	ILMN_1729749	1.625	4.96E-04

ATB vs LTB down regulated genes common to South Africa & Malawi.

S.no	Gene	Probset ID	Mean fold change	Mean P-value
1.	*NDRG2*	ILMN_2361603	2.17	1E-07
2.	*KLF12*	ILMN_1762801	2.77	1.4E-06
3.	*ANO9*	ILMN_1798679	4.05	1E-07
4.	*CD79A*	ILMN_1659227	3.80	1E-07
5.	*FCGBP*	ILMN_2302757	3.64	1E-07
6.	*GZMK*	ILMN_1710734	2.22	1E-07
7.	*CXCR3*	ILMN_1797975	2.27	1E-07
8.	*MIEF2*	ILMN_1815923	2.06	4.2E-06
9.	*CXCR5*	ILMN_2337928	2.86	1E-07
10.	*CD27*	ILMN_1688959	2.15	1E-07
11.	*RPL31P43*	ILMN_3276676	2.73	1E-07
12.	*FAM159A*	ILMN_2185675	2.63	1E-07
13.	*HPCAL4*	ILMN_1736976	2.07	1E-07
14.	*RLTPR*	ILMN_1746138	2.92	1E-07
15.	*COL9A2*	ILMN_1685122	3.48	1E-07
16.	*FAM102A*	ILMN_1745112	2.85	1E-07
17.	*DNHD1*	ILMN_1810267	2.74	1E-07
18.	*ID3*	ILMN_1732296	2.86	1E-07
19.	*TCF7*	ILMN_1676470	2.70	1E-07
20.	*LAMA5*	ILMN_1773567	2.21	6E-07
21.	*CARNS1*	ILMN_1725746	2.41	1E-07
22.	*TNFRSF25*	ILMN_1765109	2.18	1E-07

23.	*NCR3*	ILMN_2044471	2.74	1E-07
24.	*CRIP2*	ILMN_1694432	4.17	1E-07
25.	*SFI1*	ILMN_1763887	2.10	4E-05
26.	*PLXNA3*	ILMN_1719972	2.49	3E-07
27.	*HIP1R*	ILMN_2396148	2.51	2E-07
28.	*MAP4K1*	ILMN_2365111	3.03	2E-07
29.	*LRRN3*	ILMN_1773650	3.07	2E-07
30.	*CLIC5*	ILMN_1778964	2.84	2E-07
31.	*FCRLA*	ILMN_1691071	2.59	2E-07
32.	*FAM129C*	ILMN_1664063	2.17	2E-07
33.	*RTKN*	ILMN_1680591	2.21	3E-07
34.	*CD19*	ILMN_1782704	2.35	3E-07
35.	*HRK*	ILMN_2193706	4.35	4E-07
36.	*PKD1*	ILMN_2339028	2.49	2E-06
37.	*EPHA4*	ILMN_1672022	2.09	4E-07
38.	*IL11RA*	ILMN_1720024	2.38	5E-07
39.	*GPR68*	ILMN_1671142	2.49	1E-06
40.	*SPIB*	ILMN_2143314	2.63	6E-07
41.	*C2orf40*	ILMN_1676822	2.08	7E-07
42.	*SCML4*	ILMN_1747436	2.67	8E-07
43.	*EMC3-AS1*	ILMN_3289346	2.53	1E-06
44.	*NFATC3*	ILMN_1685810	2.48	9E-06
45.	*DIP2C*	ILMN_1676062	2.10	1E-06
46.	*EPHB6*	ILMN_1802646	2.16	2E-06
47.	*FCRL2*	ILMN_1665152	2.15	2E-06
48.	*OSBPL10*	ILMN_1669497	5.05	2E-06
49.	*LINC01550*	ILMN_3247639	2.18	3E-06
50.	*DTX3*	ILMN_1658677	2.50	4E-06
51.	*TLE2*	ILMN_1814917	2.64	4E-06
52.	*FLNB*	ILMN_1664922	2.14	4E-06
53.	*MATK*	ILMN_1669321	2.18	4E-05
54.	*LRRC26*	ILMN_2249018	2.19	5E-06
55.	*PCNXL2*	ILMN_2295918	2.16	5E-06
56.	*PFN1P3*	ILMN_1676016	2.15	7E-06
57.	*HAUS5*	ILMN_3247504	2.18	7E-06
58.	*LINC00926*	ILMN_3236036	2.94	7E-06
59.	*CA11*	ILMN_1743219	2.30	7E-06
60.	*PNOC*	ILMN_1676003	2.15	8E-06
61.	*AEBP1*	ILMN_1736178	2.25	8E-06
62.	*WDR86*	ILMN_1700248	2.11	1E-05
63.	*NBL1*	ILMN_1789599	2.12	1E-05
64.	*FAM153C*	ILMN_1733983	2.11	6E-05
65.	*SIRPG*	ILMN_1676780	2.40	1E-05
66.	*RTBDN*	ILMN_2384405	2.35	1E-05
67.	*EBF1*	ILMN_1778681	5.63	1E-05
68.	*WNT7A*	ILMN_1778575	2.13	2E-05
69.	*TJP3*	ILMN_1659610	2.73	3E-05
70.	*HTR3A*	ILMN_2371079	2.26	3E-05
71.	*PARM1*	ILMN_1656560	2.31	4E-05
72.	*LOC90925*	ILMN_1794927	2.69	4E-05
73.	*DKK3*	ILMN_2398159	2.16	4E-05
74.	*TNFRSF13C*	ILMN_1731742	3.63	4E-05
75.	*HABP4*	ILMN_2107991	2.72	5E-05
76.	*RNF214*	ILMN_1800420	2.23	5E-05
77.	*VPREB3*	ILMN_1700147	3.89	5E-05
78.	*BLK*	ILMN_1668277	2.81	6E-05
79.	*MSC*	ILMN_1741404	2.09	3E-04
80.	*GGTLC1*	ILMN_1680730	2.31	6E-05

81.	*SFMBT1*	ILMN_2391750	2.47	6E-05
82.	*LAMB2P1*	ILMN_3242152	2.04	6E-05
83.	*SPNS3*	ILMN_1668984	2.19	6E-05
84.	*CD8B*	ILMN_2354191	2.17	3E-04
85.	*IL23A*	ILMN_1715603	2.14	7E-05
86.	*C11orf80*	ILMN_1790637	2.70	7E-05
87.	*PAQR7*	ILMN_1746618	2.12	7E-05
88.	*LOC100133866*	ILMN_3246538	2.15	9E-05
89.	*MEGF6*	ILMN_3241441	2.15	1E-04
90.	*CEP68*	ILMN_1808500	2.24	1E-04
91.	*MS4A1*	ILMN_1776939	2.27	1E-04
92.	*LARGE*	ILMN_1662038	2.49	1E-04
93.	*CSNK1E*	ILMN_1708858	2.47	2E-04
94.	*CKB*	ILMN_1671478	2.27	2E-04
95.	*SAMD3*	ILMN_2343618	2.04	2E-04
96.	*TCL1A*	ILMN_1788841	2.62	2E-04
97.	*KRI1*	ILMN_1751395	2.05	3E-04
98.	*GOLGA8A*	ILMN_1712469	1.851852	1.93E-05
99.	*MORN3*	ILMN_1777934	1.904762	2.00E-07
100.	*PLCH2*	ILMN_2061565	1.785714	4.41E-05
101.	*GNLY*	ILMN_2256295	1.904762	1.13E-04
102.	*ZC4H2*	ILMN_3248906	1.818182	2.70E-06
103.	*PASK*	ILMN_1754858	1.960784	2.40E-06
104.	*NTN5*	ILMN_1767343	1.626016	2.25E-04
105.	*OCM2*	ILMN_3240349	1.923077	4.75E-05
106.	*TIGIT*	ILMN_2125017	1.869159	1.94E-04
107.	*BDH1*	ILMN_1799280	1.6	1.94E-05
108.	*MMP11*	ILMN_1655915	1.801802	5.80E-06
109.	*ZCCHC18*	ILMN_3214625	1.680672	5.45E-06
110.	*FBLN5*	ILMN_1664176	1.801802	9.60E-06
111.	*KIF5C*	ILMN_2212999	1.960784	1.36E-04
112.	*CCL28*	ILMN_1701347	1.818182	2.55E-04
113.	*CD8A*	ILMN_1760374	1.574803	4.52E-05
114.	*CAMTA1*	ILMN_1661940	1.818182	2.51E-05
115.	*TPM2*	ILMN_1757604	1.960784	2.42E-05
116.	*SLC29A2*	ILMN_1730809	2.061856	3.80E-05
117.	*C3orf18*	ILMN_1728581	1.851852	3.11E-05
118.	*PLXNA1*	ILMN_1791569	1.923077	4.87E-05
119.	*PDE8B*	ILMN_2301722	1.941748	2.71E-05
120.	*NEURL4*	ILMN_2407811	1.980198	4.04E-05
121.	*IFNLR1*	ILMN_1680805	1.904762	4.61E-05
122.	*GPR183*	ILMN_2168217	1.904762	8.72E-05
123.	*ZNF589*	ILMN_1654612	1.923077	5.00E-05
124.	*CNTNAP1*	ILMN_1692398	1.785714	5.96E-05
125.	*CD72*	ILMN_1723004	1.886792	5.43E-05
126.	*SNORD83B*	ILMN_3244157	1.639344	4.27E-04
127.	*WNT10B*	ILMN_1712283	1.785714	6.09E-05
128.	*BZRAP1*	ILMN_2111229	1.666667	2.20E-04
129.	*RORC*	ILMN_1771126	1.960784	1.80E-04
130.	*MIR124-2HG*	ILMN_3269119	1.6	7.09E-05
131.	*KIR3DL3*	ILMN_2082593	1.785714	1.07E-04
132.	*KIR2DL3*	ILMN_1667232	1.652893	3.62E-04
133.	*PNMA3*	ILMN_1783805	1.666667	1.17E-04
134.	*PPAN*	ILMN_1798459	1.923077	1.40E-04
135.	*AHDC1*	ILMN_1680111	1.851852	1.40E-04
136.	*TPPP3*	ILMN_1797744	1.941748	1.72E-04
137.	*SYTL2*	ILMN_2336609	1.652893	3.79E-04
138.	*APBA2*	ILMN_1723626	2	1.94E-04

139.	*ESPNL*	ILMN_2115862	1.754386	2.06E-04
140.	*OBSCN*	ILMN_1749667	1.923077	2.30E-04
141.	*KLKB1*	ILMN_1800468	1.754386	4.46E-04
142.	*CACNA1H*	ILMN_1671263	1.818182	1.70E-04
143.	*PPP2R5D*	ILMN_2359887	1.818182	2.99E-04
144.	*SMC1A*	ILMN_1652006	1.680672	6.02E-05
145.	*LOC654113*	ILMN_1813114	1.818182	2.36E-05
146.	*LOC728452*	ILMN_1722223	2.083333	1.10E-06
147.	*LOC100133930*	ILMN_3241957	1.785714	2.54E-05
148.	*LOC643695*	ILMN_1653198	1.724138	9.82E-05
149.	*LOC441528*	ILMN_1703881	1.639344	1.62E-04
150.	*LOC649917*	ILMN_3205424	1.923077	1.03E-04
151.	*LOC400464*	ILMN_1786168	1.904762	1.16E-04
152.	*LOC729985*	ILMN_1710220	1.6	5.74E-04
153.	*LOC391157*	ILMN_1802963	1.869159	1.50E-04
